# Genomic adaptations of *Vibrio campbellii* to thermal and salinity stress: insights into marine pathogen resilience in a changing ocean

**DOI:** 10.1186/s12864-025-11908-z

**Published:** 2025-08-08

**Authors:** Jiranan Pattano, Thitaporn Dechathai, Netnapa Chaichanit, Komwit Surachat, Korakot Wichitsa-nguan Jetwanna, Kanchana Srinitiwarawong, Pimonsri Mittraparp-arthorn

**Affiliations:** 1 Division of Biological Science, Faculty of Science, Prince of Songkla University, Hat Yai, Songkhla 90110 Thailand; 2 Faculty of Science, Center of Research and Innovation Development of Microbiology for Sustainability (RIMS), Prince of Songkla University, Hat Yai, Songkhla 90110 Thailand; 3 Department of Biomedical Sciences and Biomedical Engineering, Faculty of Medicine, Prince of Songkla University, Hat Yai, Songkhla 90110 Thailand; 4 Faculty of Medicine, Translational Medicine Research Center, Prince of Songkla University, Hat Yai, Songkhla 90110 Thailand; 5 Division of Computational Science, Faculty of Science, Prince of Songkla University, Hat Yai, Songkhla, 90110 Thailand

**Keywords:** Climate change, Biofilm formation, Environmental persistence, Luminous vibriosis, Osmoadaptation, Salinity adaptation, Shrimp aquaculture, Temperature stress, Thermal adaptation, *Vibrio campbellii*

## Abstract

**Background:**

Rising ocean temperatures and salinity fluctuations driven by climate change are reshaping marine microbial communities, including pathogenic *Vibrio* species. *Vibrio campbellii*, a major marine pathogen in shrimp aquaculture, needs to adapt to these environmental changes to survive and maintain virulence. However, the molecular mechanisms underlying its response to combined thermal and osmotic stress are largely unexplored.

**Results:**

This study examines the physiological responses of pathogenic *V. campbellii* strain HY01 and non-pathogenic strain ATCC BAA-1116 under combined temperature (25 °C, 30 °C, and 35 °C) and salinity (20, 30, and 60 ppt) conditions. Strain HY01 exhibited remarkable adaptability across all tested conditions, whereas ATCC BAA-1116 demonstrated reduced resilience under specific temperature-salinity combinations. Growth at 30–35 °C with elevated salinity promoted bioluminescence, swimming motility, and biofilm formation in both strains. Using transcriptomic analysis, our findings reveal that increased salinity enhances bacterial resilience under thermal stress by upregulating genes associated with metabolic pathways, oxidative phosphorylation, and ribosomal function. While elevated temperature and salinity suppress certain virulence traits (e.g., T6SS, flagellar assembly), they concurrently promote biofilm formation, enabling persistence in marine environments. Additionally, genes involved in osmoadaptation, such as those encoding compatible solutes, were highly expressed under extreme salinity. The observed shifts in gene expression highlight a coordinated regulatory network that balances cellular energy production, stress defense mechanisms, and colonization potential.

**Conclusions:**

This study provides a better understanding into the adaptive strategies of *V. campbellii* in response to thermal and osmotic stressors. These findings are particularly relevant for understanding how climate change-driven environmental shifts influence the ecology and pathogenicity of marine vibrios. Future studies should explore the functional consequences of these adaptations in shrimp-pathogen interactions, contributing to sustainable aquaculture practices.

**Supplementary Information:**

The online version contains supplementary material available at 10.1186/s12864-025-11908-z.

## Introduction

*Vibrio campbellii* is a ubiquitous marine bacterium found in various aquatic environments, including marine, estuarine, brackish, and coastal waters. It is a significant shrimp pathogen responsible for luminous vibriosis and has recently been identified as a causative agent of hepatopancreatic necrosis disease in shrimp [1, 2]. *V. campbellii* can enter shrimp rearing systems through natural seawater, live feed, and the exoskeletons of spawners. This bacterium encounters fluctuations in temperature and salinity in both oceanic and shrimp cultivation environments. The quorum sensing system in *V. campbellii* plays a critical role in regulating gene expression related to physiological behaviors such as biofilm formation, enzymatic activity, and flagellar motility [3–6], functions that are essential for bacterial adaptation, survival, and persistence in shrimp farms and marine environments.

*V. campbellii* is closely related to *V. harveyi*, a well-documented pathogen of cultured shrimp. Previous studies revealed that *V. campbellii* strains HY01 and ATCC BAA-1116 were initially misidentified as *V. harveyi* [7]. Research has demonstrated the rapid proliferation of *V. harveyi* in warm conditions [8–10]. Ocean salinity varies from 0.5 to 35 ppt, while sea surface temperatures fluctuate based on geographic location and season. Warm seawater (30 °C) with limited nutrients can negatively affect *V. harveyi* morphology and metabolic pathways but may also upregulate virulence genes [11]. Conversely, *V. harveyi* can enter a viable but non-culturable (VBNC) state under low temperatures (4 °C), regaining vegetative growth when conditions become favorable [12]. Similarly, varying salt concentrations can trigger the VBNC state in *V. harveyi* [13]. Although *V. harveyi* and *V. campbellii* share genotypic and phenotypic similarities, their specific adaptive responses to temperature and salinity remain insufficiently characterized. Understanding the environmental factors that influence *V. campbellii* adaptation is essential for predicting the risk of vibriosis outbreaks in shrimp aquaculture, particularly in the context of ocean warming.

To date, no studies have systematically investigated the physiological responses of *V. campbellii* strains to different temperature and salinity conditions. Therefore, this study aims to compare the phenotypic and genotypic profiles of two *V. campbellii* strains and elucidate their adaptive mechanisms under elevated temperatures with varying salinities. We utilized the pathogenic strain HY01 and non-pathogenic strain ATCC BAA-1116, each isolated from distinct geographical regions. These strains were cultured at different salinities (20, 30, and 60 ppt) and incubated at temperatures mimicking warm (25 °C), very warm (30 °C), and hot (35 °C) climates. We hypothesized that the two strains would exhibit distinct phenotypic patterns under these conditions. The findings of this study provide valuable insights into the adaptive strategies of pathogenic *V. campbellii*, contributing to the understanding of its survival mechanisms and the potential risks of vibriosis outbreaks in shrimp aquaculture under climate change scenarios.

## Materials and methods

### Bacterial strains and culture conditions

A pathogenic *V. campbellii* strain HY01, isolated from dead shrimp affected by luminous vibriosis in Thailand [14], and non-pathogenic strain ATCC BAA-1116, isolated from a marine environment in the United States [15], were used in this study. These strains were cultured in Luria–Bertani broth (LB) supplemented with artificial sea salt (Marinium Reef sea salt, Thailand) (LBS) to achieve final salinity levels of 20 ppt, 30 ppt, or 60 ppt, and incubated at 25 °C, 30 °C, or 35 °C. The pH of LBS broth or agar was adjusted to pH 8 to reflect the average pH conditions of global seawater and shrimp ponds [16, 17]. In all experiments, cultures in the mid-log exponential growth phase were diluted to a final cell concentration at 10^5^ CFU/ml.

### Growth and bioluminescence kinetic assays

The two *V. campbellii* strains were cultured in 96-well clear plates containing LBS at 20 ppt, 30 ppt, or 60 ppt. Bioluminescent measurements were conducted in 96-well white microplates and incubated in a microplate reader (LUMIstar, BMG LabTech, Germany) at 25 °C, 30 °C, or 35 °C with orbital shaking. Growth and luminescence were recorded at wavelengths of 600 nm and 490 nm, respectively.

### Swimming motility assay

The motility assays were performed using 0.3% LBS agar with 20 ppt, 30 ppt, or 60 ppt salinity LBS. A-1 μl aliquot of bacterial cultures was inoculated at the center of soft agar plates and incubated overnight at 25 °C, 30 °C, or 35 °C. The diameters of motility zones were measured at 16 h and 24 h. Motility extent was quantified using the following formula:

Plate coverage (%) = (Diameter of the motility (mm)/49 mm of inner glass plate diameter) × 100%

### Biofilm formation assay

#### Quantitation of biofilm biomass

The biofilm-forming ability of *V. campbellii* strains was assessed using crystal violet (CV) assay [18]. Briefly, cultures were grown in LBS supplemented with 20 ppt, 30 ppt, or 60 ppt salinity and incubated at 25 °C, 30 °C, or 35 °C for 48 h. After incubation, planktonic cells were removed, and wells were washed with distilled water and stained with 0.1% CV. The CV-stained biofilms were solubilized with 95% ethanol, and absorbance was measured at 570 nm.

#### Microscopic visualization

To assess biofilm formation, *V. campbellii* strains were cultured in 24-well plates containing 60 ppt salinity LBS and glass slides at 25 °C, 30 °C, or 35 °C. Mature biofilms were stained with fluorescein isothiocyanate-conjugated concanavalin A (FITC-ConA) and propidium iodide (PI) using FluoroTag™ conjugation kit (Sigma–Aldrich, USA) following the previous protocol with slight modification [19, 20]. Biofilms were visualized using CLSM (LSM800 ZEISS, Germany).

### Surface colonization assay

White leg shrimp shells were used as a natural substrate for biofilm formation by *V. campbellii*. Biofilm structures on shrimp shells were examined using SEM [21]. Shells were cut into small pieces and sterilized with 50% formaldehyde solution before exposure to bacterial suspensions in 24-well plates containing 60 ppt salinity LBS. Samples were incubated at 25 °C, 30 °C, or 35 °C without shaking. After incubation, non-adherent cells were removed, and surface-attached biofilms were fixed with 2.5% glutaraldehyde (Sigma Aldrich, USA) at 4°C for 2 h. The samples were then dehydrated through an ethanol gradient, gold coated, and examined under SEM.

### RNA isolation, sequencing and RNA-seq data analysis

Strain HY01 was cultured in LBS at 30 ppt or 60 ppt salinity and incubated at 30 °C or 35 °C until reaching mid-exponential growth phase (5–6 h). Cells were collected via centrifugation (8,000 rpm, 3 min), and total RNA was extracted using the Direct-zol RNA Microprep Kits (Zymo Research, USA). The purified RNA was preserved in RNA stabilization tubes (GENEWIZ, USA) before library preparation and sequencing. RNA sequencing was performed using NovaSeq 6000 platform. Read quality was assessed using FastQC (v0.74), and adaptor trimming and low-quality read removal were conducted with Fastp (v0.24.0). Processed reads were aligned to the draft genome assembly of *V. campbellii* strain HY01 using HISAT2 (v2.2.1), and gene transcripts were assembled with Stringtie (v2.2.3). The draft genome assembly of *V. campbellii* strain HY01 (NCBI accession number SCEL00000000) was used as the reference and is publicly accessible in GenBank. Differentially expressed genes (DEGs) were identified using edgeR (v3.62.1) and limma (v4.4.1) in RStudio (v4.4.2). DEGs were classified into 3 groups based on fold change (FC) values at an adjusted FDR *p*-value ≤ 0.05 (upregulated genes: log_2_ FC ≥ 2; downregulated genes: log_2_ FC ≤ − 2; no different expression: − 2 < log_2_ FC < 2). Visualization of DEGs was performed using pheatmap (v1.0.12) and ggVennDiagram (v1.5.2) in R. Volcano plots were generated using SRplot (https://www.bioinformatics.com.cn/en). Functional enrichment analysis was conducted using ShinyGO 0.81 (http://bioinformatics.sdstate.edu/go/), and transcripts were mapped to the NCBI database for Clusters of Orthologous Groups (COGs) classification. Gene networks visualization was performed using STRING v12.0 (https://string-db.org/).

### Reverse transcription-quantitative PCR (RT-qPCR)

RT-qPCR validation was performed on selected genes involved in adaptive responses to temperature and salinity stress. Primer sequences are listed in Table S1. Strain HY01 was cultured and processed under the same RNA isolation conditions. Total RNA was reverse transcribed to complementary DNA (cDNA) using the FIREScript RT cDNA synthesis kit (Solis Biodyne, Estonia). qPCR reactions were carried out using Luna Universal qPCR Master Mix (NEB, USA) with 10 µM forward and reverse primers, and 10 ng cDNA. Then, the reaction was amplified using LineGene 9600 Plus, Real–Time PCR Detection System (Bioer, USA). The fold change of gene expression was calculated using the 2^−(∆∆Ct)^ method, normalized to the 16S rRNA reference gene. Correlation between RNA-seq and qRT-PCR results was evaluated using Pearson correlation, and plots were generated in ggplot2 (v3.5.1) in R. The Pearson’s R correlation coefficients were interpreted according to the criteria reported by Schober et al. [22].

### Statistical analysis

Data were represented as mean ± standard deviation. Normality, homogeneity, and factor interactions were assessed using SPSS (v20.0). The Shapiro–Wilk test confirmed normal distribution, while Levene’s test indicated variance heterogeneity (*p* < 0.05). Aligned Rank Transform ANOVA was applied to assess factor interactions, followed by multiple comparisons using the Games-Howell post-hoc test.

## Results

### Temperature and salinity affected the growth kinetics of *Vibrio campbellii*

**Fig. 1:**
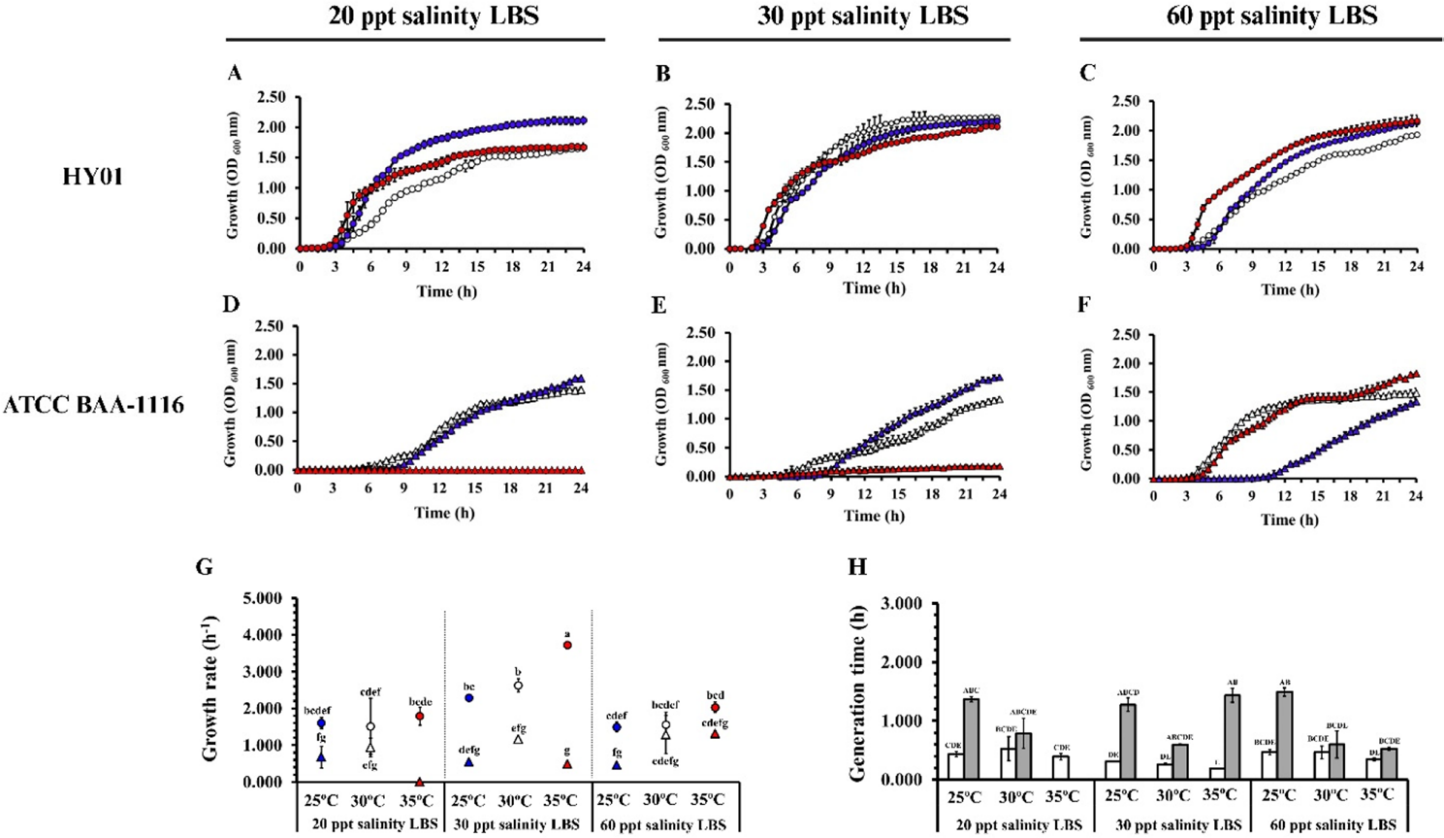
Effect of combined temperature and salinity on *Vibrio campbellii* growth. **A–F** Growth curves of *V. campbellii* strains HY01 (circle) and ATCC BAA-1116 (triangle) in Luria–Bertani broth supplemented with artificial seawater (LBS) at 25  °C (, ), 30 °C (, ), and 35 °C (, )  °C under different salinity conditions. **G** Growth rates and **H** generation times were calculated from the exponential growth phases under various temperature-salinity conditions. Generation times of HY01 and ATCC-BAA1116 are represented by white and gray bars, respectively. Multiple comparisons were conducted using Games-Howell post hoc analysis due to variance heterogeneity (*p* < 0.05, *n* = 3 per group). Different lowercase letters indicate statistically significant differences

The effect of temperature and salinity on the growth kinetics of *V. campbellii* was systematically assessed. Growth profiles of strains HY01 and ATCC BAA-1116 were evaluated under nine conditions, combining three temperatures (25 °C, 30 °C, or 35 °C) with three salinities (20 ppt, 30 ppt, or 60 ppt). The results highlighted significant effects of temperature and salinity on the entry into the exponential phase (Fig. 1A–F), growth rates (Fig. 1G), and generation times (Fig. 1H) for both strains. Strain HY01 exhibited remarkable adaptability across all conditions. No significant differences in growth were observed across difference temperatures within each salinity group, except for 30 ppt LBS at 35 °C, where HY01 displayed the highest growth rate and shortest generation time. Conversely, strain ATCC BAA-1116 demonstrated resilience under specific conditions. Both 25 °C and 35 °C conditions resulted in lower biomass yields, with this effect being more pronounced at higher temperatures combined with lower salinity. Interestingly, lower salinity extended the lag phase of ATCC BAA-1116, particularly at 30 °C, where the lag phase duration was extended to 6 h at 20 ppt, compared to 5 h at 30 ppt and 3 h at 60 ppt. Furthermore, strain ATCC BAA-1116 failed to grow at 35 °C; however, increasing salinity significantly enhanced survival and facilitated entry into the exponential phase. The interaction between bacterial strains, temperature, and salinity significantly affected growth rate and generation time (Table 1).

**Table 1:** Aligned Rank Transform ANOVA analysis of non-parametric variances for three-way interactions among factors (*Vibrio campbellii* strains, temperature, and salinity) with the dependent variables (growth rate, generation time, bioluminescence, and biofilm formation)

Factors	df	Growth rate		Generation time		Bioluminescence		Biofilm formation	
		Fvalue	*P* value	F value	*P* value	F value	*P* value	F value	*P* value
Strain	1	85.459	< 0.001*	47.090	< 0.001*	60.211	< 0.001*	84.559	< 0.001*
Salinity	2	10.360	< 0.001*	1.216	0.309	12.560	< 0.001*	31.798	< 0.001*
Temperature	2	10.230	< 0.001*	4.760	0.015*	30.826	< 0.001*	29.411	< 0.001*
Strain × Salinity	2	25.862	< 0.001*	5.553	0.008*	31.327	< 0.001*	6.321	0.005*
Strain × Temperature	2	6.468	0.004*	5.602	0.008*	9.879	< 0.001*	14.014	< 0.001*
Salinity × Temperature	4	0.639	0.639	3.797	0.012**	21.519	< 0.001*	104.526	< 0.001*
Strain × Salinity × Temperature	3	6.029	0.002*	3.649	0.022*	19.584	< 0.001*	47.091	< 0.001*

“*” represents a statistically significant difference at *p* < 0.05

### Bioluminescence of *Vibrio campbellii* is influenced by culture conditions

**Fig. 2:**
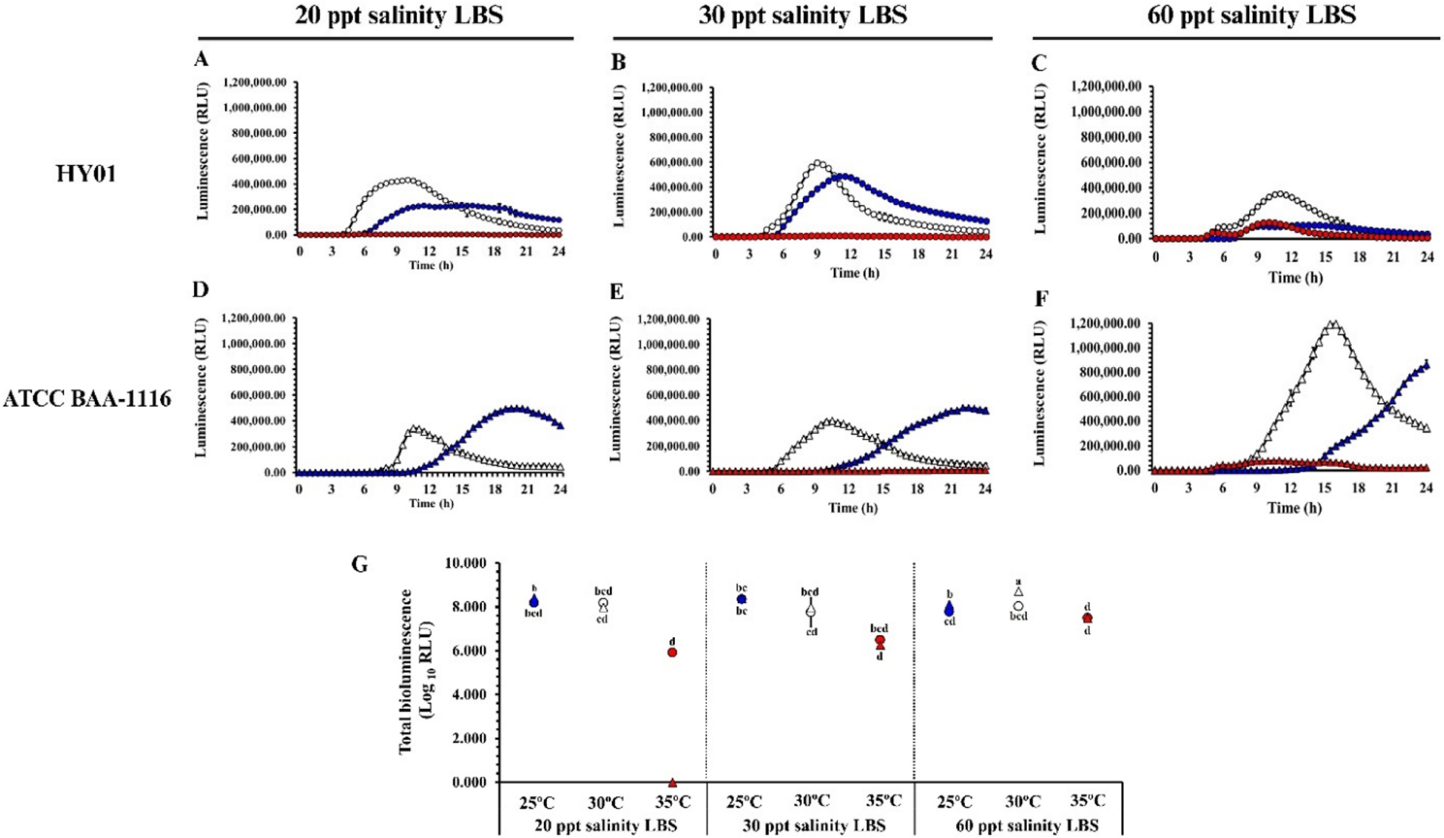
Effect of combined temperature and salinity on bioluminescence of *Vibrio campbellii*. **A–F** Bioluminescence of *V. campbellii* strains HY01 (circle) and ATCC BAA-1116 (triangle) over 24 h at 25 °C (, ), 30 °C (, ), and 35 °C (, )  °C under different salinity conditions. **G** Total bioluminescence (log_10_ RLU) calculated from the area under the curves. Multiple comparisons were conducted using Games-Howell post hoc analysis (*p* < 0.05, *n* = 3 per group). Different lowercase letters denote statistically significant differences

Both HY01 and ATCC BAA-1116 exhibited similar luminescence patterns across growth phases, but the intensity varied depending on culture conditions (Fig. 2A–F). Luminescence peaked during the exponential phase and decreased upon entering the stationary phase. Elevated temperatures (35 °C) led to reduced luminescence intensity, where increased salinity resulted in slightly higher light emissions. Notably, ATCC BAA-1116 exhibited its highest luminescence when grown in 60 ppt salinity LBS at 30 °C (1.19 × 10^6^ RLU, 18 h) compared to other conditions. These findings suggest that elevated salinity may enhance survival and bioluminescence activity under high-temperature conditions. Analysis of total luminescence revealed that ATCC BAA-1116 produced the highest luminescence at 60 ppt salinity and 30 °C (Fig. 2G). Statistical analysis confirmed that bioluminescence was significantly influenced by interactions between salinity and temperature (Table 1).

### Swimming motility of *Vibrio campbellii* is altered by temperature and salinity

**Fig. 3:**
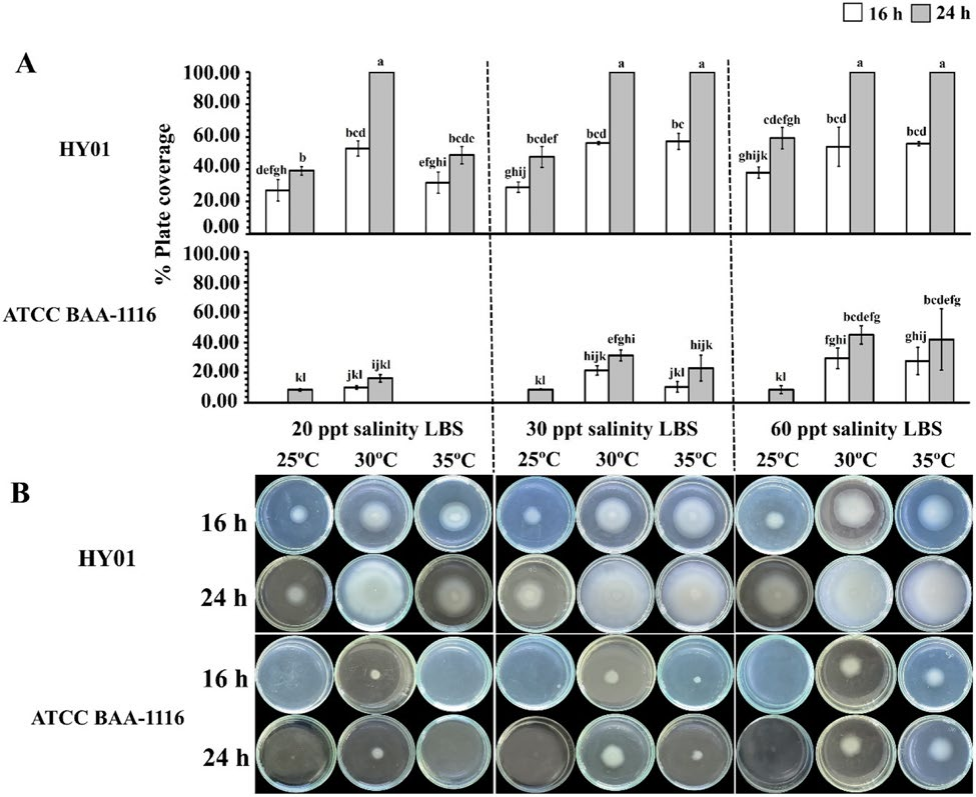
Effect of combined temperature and salinity on swimming motility of *Vibrio campbellii*.** A** Swimming motility was assessed by measuring colony expansion across a 49 mm plate at different incubation times. **B** Representative images of motility plates showing *V. campbellii* HY01 and ATCC BAA-1116 after 16 h and 24 h of incubation. Multiple comparisons were conducted using Games-Howell post hoc analysis (*p* < 0.05, *n* = 3 per group). Different lowercase letters denote statistically significant differences

Swimming motility, driven by the polar flagellum, is a crucial virulence factor facilitating bacterial dispersal and biofilm formation. Both strains were cultured on semi-solid LBS agar at varying salinities and temperatures. Strain HY01 exhibited the greatest motility at 20 ppt salinity and 30 °C, as well as at 30 ppt and 60 ppt salinities at 30 °C and 35 °C (Fig. 3A). This resulted in rapid colony expansion across the plate within 24 h (Fig. 3B). Conversely, ATCC BAA-1116 colonies were not visible until 16 h of incubation at 25 °C, with motility first detected at 24 h across all salinity groups (Fig. 3B). Notably, increasing salinity significantly enhanced motility in both strains at higher temperatures. Statistical analysis revealed that motility was significantly influenced by bacterial strains, salinity, and temperature. Interactions among incubation time, strain, and temperature showed significant effects, whereas the interaction of all four factors (strains × incubation time × temperature × salinity) did not exhibit statistically significant difference (Table 2).

**Table 2:** Aligned Rank Transform ANOVA analysis of non-parametric variances for interactions among four factors, including bacterial strains, incubation time, temperature, and salinity, with swimming motility in *Vibrio campbellii*

Factors	df	Swimming motility (% plate coverage)	
		F value	*P* value
Strain	1	199.122	< 0.001*
Incubation time	1	172.113	< 0.001*
Salinity	2	20.477	< 0.001*
Temperature	2	81.492	< 0.001*
Strain × Incubation time	1	113.737	< 0.001*
Strain × Salinity	2	13.724	< 0.001*
Strain × Temperature	2	33.904	< 0.001*
Incubation time × Salinity	2	2.416	0.097
Incubation time × Temperature	2	32.085	< 0.001*
Salinity × Temperature	4	19.400	< 0.001*
Strains × Incubation time × Salinity	2	0.610	0.546
Strains × Incubation time × Temperature	2	19.354	< 0.001*
Strain × Salinity × Temperature	3	3.317	0.025*
Incubation time × Salinity × Temperature	4	1.988	0.106
Strains × Incubation time × Salinity × Temperature	3	0.252	0.860

“*” represents a statistically significant difference at *p* < 0.05

### Biofilm formation in *Vibrio campbellii* is influenced by temperature

**Fig. 4:**
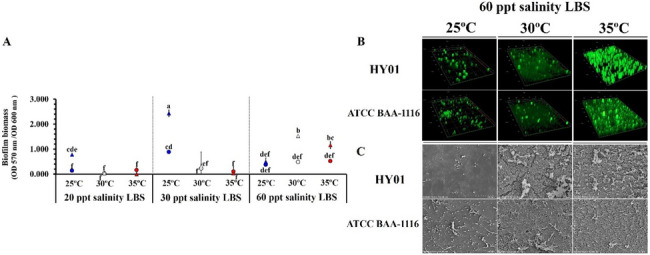
Biofilm formation of *Vibrio campbellii* strains under different conditions. **A** Quantification of biofilm biomass formed by *V. campbellii* strains HY01 (circle) and ATCC BAA-1116 (triangle) at 25 °C (blue), 30 °C (white), and 35 °C (red) under different salinity conditions. **B** Confocal laser scanning microscopy (CSLM) images of biofilms formed at 60 ppt under different temperatures (25 °C, 30 °C, and 35 °C).** C** Scanning electron microscopy (SEM) images showing *V. campbellii* biofilm formation on shrimp shells at different temperatures. Statistical significance was determined using Games-Howell post hoc analysis (*p* < 0.05, with *n* = 3 replicates). Different lowercase letters represent statistically significant differences

The CV assay was performed to examine the biofilm biomass of *V. campbellii* strains cultured under varying temperature and salinity conditions. The highest biofilm biomass in both strains was observed at 25 °C across all salinity levels. When cultured at 20–30 ppt, biofilm formation significantly decreased at 30 °C and 35 °C. However, at 60 ppt salinity, increasing temperatures enhanced biofilm formation in both strains, particularly in the pathogenic strain HY01, which exhibited greater biofilm biomass than the non-pathogenic strain (Fig. 4A). Consequently, 60 ppt salinity was selected for further examination of biofilm quantities and structural characteristics under temperature-induced stress conditions. *V. campbellii* strains HY01 and ATCC BAA-1116 were allowed to adhere to glass slides (abiotic surfaces) and white leg shrimp shells (biotic surfaces) in LBS medium (60 ppt salinity) at 25 °C, 30 °C, or 35 °C. To indirectly quantify biofilms, exopolysaccharide production was visualized using FITC-ConA (green fluorescence), while cell viability was assessed using PI staining (red fluorescence). CLSM images revealed extensive green fluorescence at 30 °C and 35 °C, indicating robust biofilm formation. Conversely, minimal fluorescence was observed under 25 °C (Fig. 4B). Additionally, SEM imaging demonstrated complex and dense biofilm structure at 30 °C and 35 °C with 60 ppt salinity, whereas significantly lower cell attachment and minimal biofilm formation were detected at 25 °C (Fig. 4C). CLSM and SEM analyses confirmed that high salinity (60 ppt) enhanced *V. campbellii* biofilm formation on both abiotic and biotic surfaces under elevated temperatures. Statistical analysis indicated that temperature, salinity, and strain type significantly influenced biofilm formation. These results suggest that increased salinity not only promotes growth and bioluminescence but also enhances swimming motility and biofilm formation in *V. campbellii* strains under high-temperature conditions.

### Differentially expressed genes of pathogenic *Vibrio campbellii* are primarily affected by temperature and salinity

**Fig. 5:**
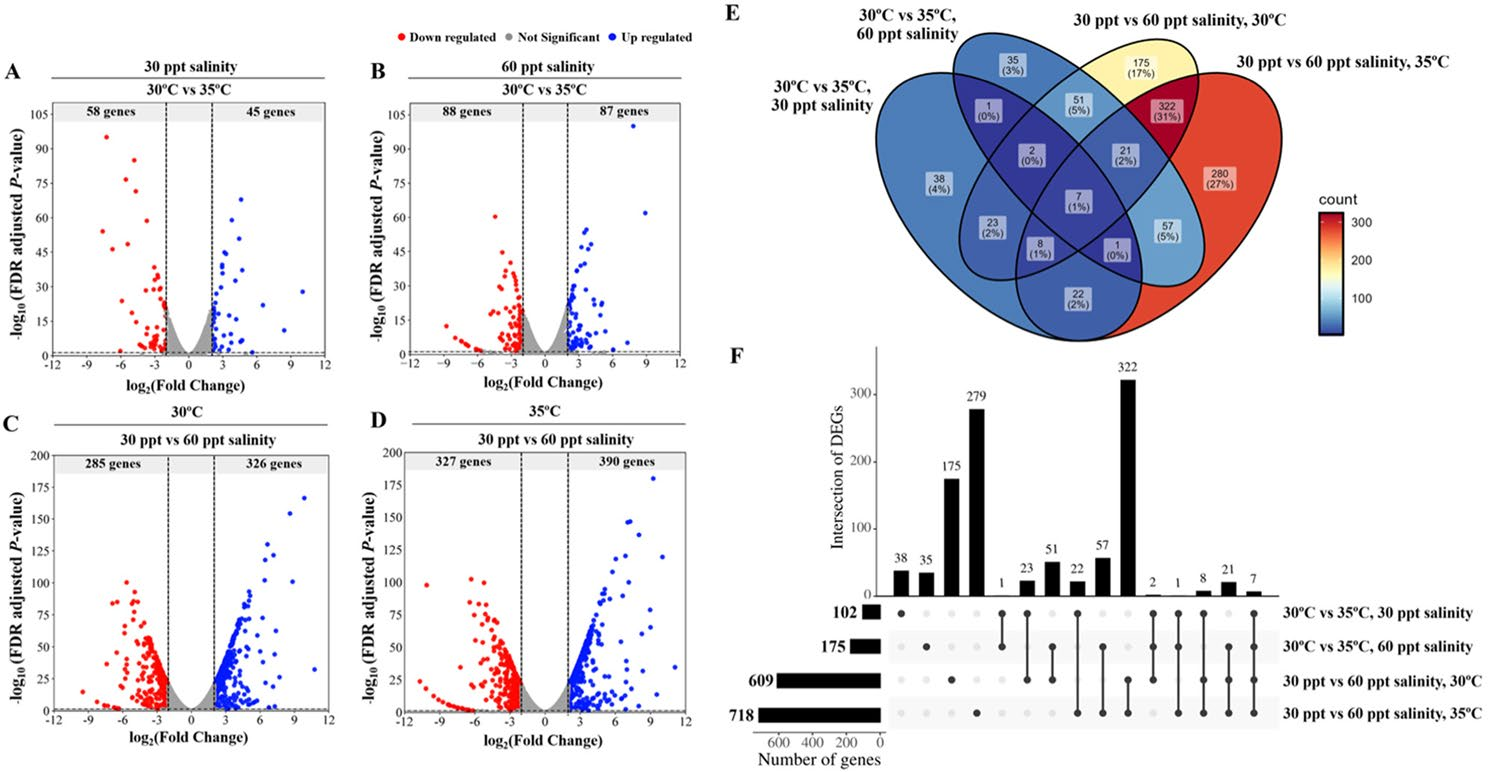
Differentially expressed genes (DEGs) in *Vibrio campbellii* HY01 under combined temperature and salinity conditions. Volcano plot depicts the DEGs in *V. campbellii* strain HY01 comparing different experimental conditions. **A** 30 °C versus 35 °C under 30 ppt salinity LBS, **B** 30 °C versus 35 °C under 60 ppt salinity LBS, **C** 60 ppt salinity LBS versus 30 ppt salinity LBS at 30 °C, **D** 60 ppt salinity LBS versus 30 ppt salinity LBS at 35 °C. The x-axis represents log2 fold change, while the y-axis shows the -log_10_ of the false discovery rate (FDR)-adjusted *p*-value. **E** Venn diagram illustrating the intersection of DEGs across four comparisons, with color intensity (red to blue) representing high to low gene numbers. **F** Bar plot displaying the number of DEGs at intersections across comparisons. Gene functions and pathways related to DEGs are detailed in the Results and Discussion sections

The phenotypic analyses demonstrated the greater adaptability of HY01 under high-temperature and high-salinity conditions. To further investigate transcriptomic responses to temperature and salinity stress, RNA-sequencing (RNA-seq) was performed on pathogenic strain HY01. RNA-seq datasets were categorized into two comparison groups: temperature (30 °C vs. 35 °C at both 30 ppt and 60 ppt salinity) and salinity (30 ppt vs. 60 ppt at both 30 °C and 35 °C), with 30  °C and 30 ppt considered as the optimal temperature and salinity conditions, respectively. The results revealed that both temperature and salinity significantly influenced differentially expressed genes (DEGs) in *V. campbellii* (Fig. 5A–D and Table S2).

Comparing growth at 30 °C and 35 °C under 30 ppt salinity, 103 DEGs were identified, including 45 upregulated and 58 downregulated genes. Upregulated genes were primarily associated with type secretion systems (TSS), transporters, and stress responses (e.g., heat shock proteins and phage shock proteins), while downregulated genes were largely involved in oxidative phosphorylation and cellular metabolism. Under 60 ppt salinity, 175 DEGs were detected, consisting of 87 upregulated and 88 downregulated genes. Upregulated DEGs were associated with cellular metabolism, ribosome function, TSS, and transporters, while flagellar construction and motility-related genes were downregulated. In the salinity comparisons at 30 °C, 611 DEGs were identified (326 upregulated, 285 downregulated). These DEGs were associated with cellular metabolism, TSS, transporters, and oxidative phosphorylation. At 35 °C with 60 ppt salinity, 717 DEGs were detected (390 upregulated, 327 downregulated), with most upregulated genes involved in metabolism, oxidative phosphorylation, bioluminescence, compatible solute uptake systems, and ribosome function. Notably, 33 ribosomal protein genes were significantly upregulated under extreme salinity and temperature conditions. Conversely, genes encoding flagellar construction, cellular components, and metabolic processes were downregulated at 35 °C with 60 ppt salinity compared to 30 ppt salinity. Interestingly, two cold-shock protein genes were unexpectedly upregulated under extreme conditions.

**Fig. 6:**
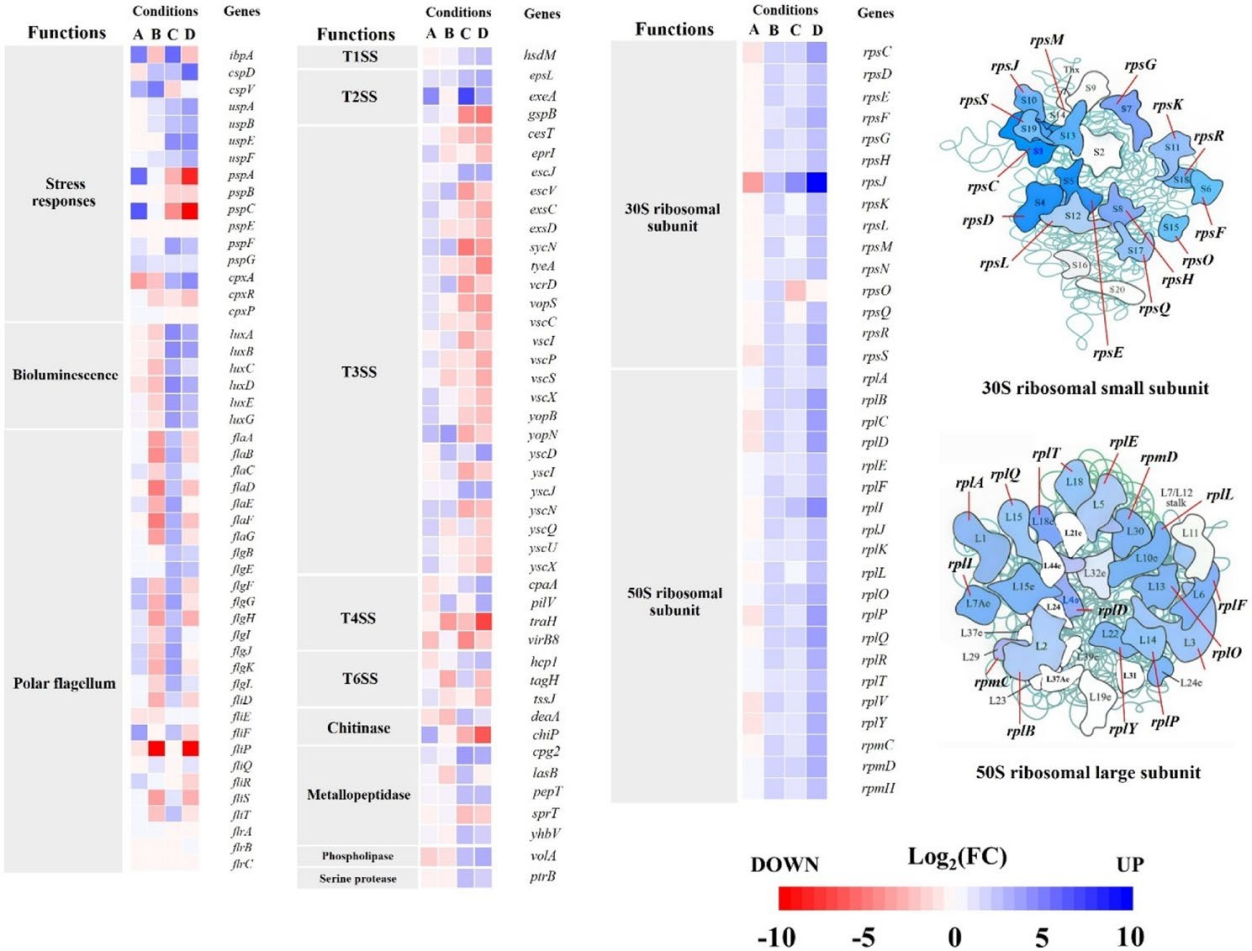
Heatmap representation of differentially expressed genes (DEGs) in *Vibrio campbellii* under combined temperature and salinity conditions. The heatmap illustrates transcript levels of DEGs associated with stress response, bioluminescence, polar flagella motility, virulence (secretion systems), enzymatic proteins, and ribosomal assembly. DEGs are categorized into four groups: **A** temperature comparison (30 °C vs. 35 °C) under 30 ppt salinity, **B** 30 °C versus 35 °C under 60 ppt salinity, **C** salinity comparison (30 ppt vs. 60 ppt) at 30 °C, and **D** 30 ppt versus 60 ppt at 35 °C. The color gradient (red to blue) indicates low to high gene expression. Specific gene functions are detailed in the Results and Discussion sections

**Fig. 7:**
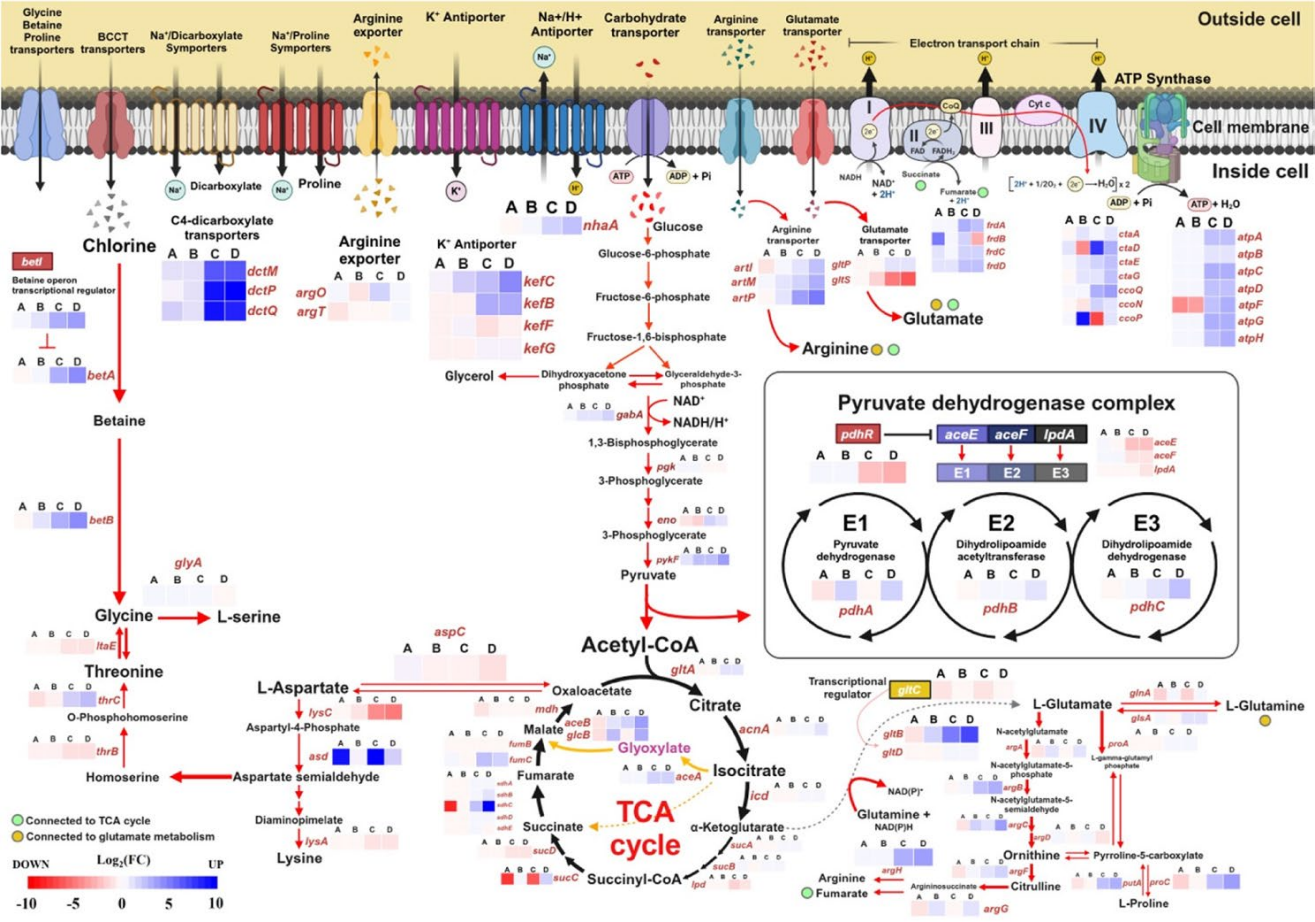
Metabolic pathways and oxidative phosphorylation related to differentially expressed genes (DEGs) of *Vibrio campbellii* under combined temperature and salinity conditions. DEGs associated with metabolic pathways are visualized using a color gradient map (red to blue, representing low to high gene expression). DEGs are categorized into: **A** temperature comparison (30 °C vs.35 °C) under 30 ppt salinity, **B** 30 °C vs.35 °C under 60 ppt salinity, **C** salinity comparison (30 ppt vs. 60 ppt) at 30 °C, and **D** 30 ppt versus 60 ppt at 35 °C. Specific genes and functions are described in the Results and Discussion sections and Table S4. Pathways were generated using BioRender.com

Across all RNA-seq datasets, seven DEGs were identified as overlapping, associated with motility, cell membrane integrity, transporters, ribosome function, and transcriptional regulation (Fig. 5EF, and Table S3). The largest intersection of DEGs (322 genes) was observed in the salinity comparison groups at different temperatures (30 ppt vs. 60 ppt at 30 °C and 35 °C), with genes linked to osmoadaptation, cell membrane functions, and bioluminescence (Figs. 6 and 7).

### Functional analysis of differentially expressed genes

Functional enrichment analysis of DEGs was conducted using Gene Ontology (GO), KEGG, and COG databases (Fig. S1 and Table 3). GO analysis classified DEGs into three domains: biological process (BP), cellular component (CC), and molecular function (MF). In the temperature comparisons (30 °C vs. 35 °C at 30 ppt and 60 ppt salinity), significant enrichment was observed in BP and CC domains, while no enrichment was observed in MF. Within the BP domain, genes related to cellular metabolism and energy production were enriched at 30 °C versus 35 °C under 30 ppt salinity, whereas genes associated with cellular processes, flagellar function, and localization were enriched at 30 °C versus 35 °C under 60 ppt salinity. The CC domain contained the highest number of DEGs, with 32 genes classified at 30 °C versus 35 °C under 30 ppt salinity and 40 genes at 60 ppt salinity (Fig. S1 A-B). In the salinity comparisons, a larger number of GO- enriched categories were identified (Fig. S1 E–F), with many DEGs involved in metabolism and cellular structures. Notably, ribosome-related GO terms were highly enriched in the 30 ppt versus 60 ppt salinity comparison at 35 °C. Both temperature and salinity influenced COG classifications of DEGs (Fig. S1 C-D, Fig. S1 G-H). Amino acid transport and metabolism (COG category E) was the most abundant across all conditions. Notably, flagella assembly-related genes were highly downregulated at 30 °C versus 35 °C under 60 ppt salinity, while genes related to ribosomal structure and translation (COG category J) were predominantly upregulated at 30 ppt versus 60 ppt under 35 °C. KEGG pathway analysis identified five significantly enriched pathways (Table 3). Oxidative phosphorylation (vha00190) was the dominant pathway across three comparisons: 30 °C versus 35 °C at 30 ppt salinity, and 30 ppt versus 60 ppt salinity at both 30 °C and 35 °C. Flagellar assembly (vha02040) was the most downregulated pathway at 30 °C versus 35 °C under 60 ppt salinity, while ribosomal-associated pathways (vha03010) were most significantly enriched at 30 ppt versus 60 ppt salinity under 35 °C. High enriched genes encoding proteins related to metabolism and oxidative phosphorylation showed a co-interactive network, suggesting systematic coordination under combined temperature and salinity conditions (Fig. S2).

**Table 3:** KEGG pathway enrichment analysis of differentially expressed genes (DEGs) in *Vibrio campbellii* under combined temperature and salinity conditions

KEGG ID	KEGG Pathway	*P*-value	FDR adjusted P-value	Number of genes	
				Up-regulated genes	Down-regulated genes
30 °C vs 35 °C, 30 ppt salinity					
vha00190	Oxidative phosphorylation	4.06 × 10^–5^	0.0054	1	4
30 °C vs 35 °C, 60 ppt salinity					
vha02040	Flagellar assembly	8.30 × 10^–10^	1.11 × 10^–7^	1	9
30 ppt vs 60 ppt salinity, 30 °C					
vha00190	Oxidative phosphorylation	3.91 × 10^–9^	5.24 × 10^–7^	11	4
vha01100	Metabolic pathways	1.56 × 10^–7^	1.04 × 10^–5^	51	26
vha00780	Biotin metabolism	0.00025	0.0112	5	1
30 ppt vs 60 ppt salinity, 35 °C					
vha03010	Ribosome	9.40 × 10^–26^	1.26 × 10^–23^	33	0
vha00190	Oxidative phosphorylation	1.74 × 10^–10^	1.17 × 10^–8^	14	4
vha01100	Metabolic pathways	8.92 × 10^–6^	4.00 × 10^–4^	62	22

### Dual-stress conditions affect the expression of general stress-responsive genes in *Vibrio campbellii*

Differential gene expression analysis under salinity stress revealed the upregulation of several genes encoding universal stress proteins (USPs), particularly in the salinity comparison group (Fig. 6 and Table S2). Notably, *uspE* exhibited strong upregulation, with log_2_FC values of 2.68 at 30 °C and 2.93 at 35 °C. In contrast, *uspA* (2.17 log_2_FC) and *uspB* (2.09 log_2_FC) were upregulated only under the 35 °C condition. No significantly differential expression of USP-related genes was observed in the temperature comparison group.

Conversely, genes encoding phage shock proteins (PSPs) were predominantly responsive to thermal stress. Both *pspA* and *pspC* were highly upregulated under elevated temperature, with log_2_FC values of 3.56 and 4.11, respectively. However, these genes were either not expressed or were strongly downregulated under salinity stress. Specifically, in the 35 °C salinity comparison group, *pspA* and *pspC* were downregulated with log_2_FC values of − 5.04 and − 6.30, respectively. Additionally, genes belonging to the two-component CpxRA regulatory system were significantly induced under salinity stress at both temperatures. The sensor kinase gene *cpxA* showed upregulation, with log_2_FC values of 2.03 at 30 °C and 2.89 at 35 °C. In contrast, no significant expression changes were detected for *cpxA* under thermal stress.

### Influence of temperature and salinity on heat- and cold-shock genes regulation

Several temperature-responsive genes, particularly those encoding heat shock proteins (HSPs) and cold shock proteins (CSPs), were significantly upregulated in response to increased temperature and salinity (Fig. 6 and Table S2). Under optimal salt concentration at 35 °C, *V. campbellii* strain HY01 exhibited a strong stress response, as evidenced by upregulation of *ibpA* (3.28 log_2_FC), which encodes a small heat shock protein (sHSP). Interestingly, *ibpA* expression was not only induced by a minor temperature increase (from 30 °C to 35 °C) but was also upregulated at 60 ppt salinity at 30 °C (3.40 log_2_FC). Additionally, at 35 °C, the cold shock protein. Gene *cspV* was upregulated under 60 ppt salinity (3.16 log_2_FC), while no expression was detected at 30 ppt salinity. Similarly, *cspD* was significantly upregulated at 35 °C under 60 ppt salinity (3.67 log_2_FC), yet remained unchanged at 30 °C.

### Effect of salinity stress on osmoprotectant-related metabolic pathways

Under salinity stress, DEG analysis revealed the upregulation of several transporter genes as a primary adaptive response to maintain ion homeostasis (Fig. 7; Table S2 and S4). These included potassium/proton antiporters *kefB* (3.48 log_2_FC) and *kefC* (4.33 log_2_FC), as well as the sodium/proton antiporter *nhaA* (2.02 log_2_FC).

In parallel, multiple metabolic pathways exhibited a coordinated gene expression network involved in the synthesis of osmoprotectants (e.g., intermediate precursors, compatible solutes, and amino acids) and the generation of cellular energy to enhance survival and adaptability in *V. campbellii*. This was supported by the interconnected DEG network related to carbohydrate and amino acid biosynthesis pathways (Fig. S2). KEGG pathway enrichment further confirmed that metabolic pathways (vha01100) were predominantly activated under high-salinity conditions (Table 3). Overlapping DEGs from both salinity comparisons (30 °C and 35 °C) were primarily associated with metabolic functions.

Key genes in oxidative metabolism pathways, including glycolysis, the pyruvate pathway, and TCA cycle, were upregulated. Notable examples include phosphoglycerate mutase *gpmI* (2.12 log_2_FC), pyruvate kinase II *pykF* (3.02 log_2_FC), succinate dehydrogenase cytochrome b subunit *sdhC* (9.00 log_2_FC), and fumarase C *fumC* (2.20 log_2_FC). Within the glyoxylate shunt of TCA cycle, *aceB* (malate synthase; 3.43 log_2_FC) and *glcB* (malate synthase G; 3.41 log_2_FC) were also upregulated. Despite the upregulation of many oxidative metabolism-related genes, some genes were downregulated, possibly reflecting regulatory fine-tuning to enhance TCA cycle efficiency. For example, *pdhR*, a transcriptional repressor of the pyruvate dehydrogenase complex, was downregulated (-2.68 log_2_FC). The increased activity of the TCA cycle activity was further supported by the elevated expression of genes encoding TRAP transporters responsible for importing C4-dicarboxylates intermediates: *dctP* (10.38 log_2_FC), *dctQ* (9.24 log_2_FC), and *dctM* (7.05 log_2_FC).

Genes involved in the biosynthesis of compatible solutes, key osmolytes for counteracting high osmotic pressure, were notably upregulated across several metabolic pathways. In the glutamate biosynthesis pathway, *gltB* (glutamate synthase large subunit) showed strong expression (6.47 log_2_FC), while *gltS* (glutamate transporter) was significantly downregulated (-5.67 log_2_FC). In the proline biosynthesis pathway, *proC* (pyrroline-5-carboxylate reductase; 3.57 log_2_FC), and *putA* (proline dehydrogenase/P5C dehydrogenase; 2.63 log_2_FC) were upregulated. Similarly, in arginine metabolism, genes such as *argH* (argininosuccinate lyase; log_2_FC of 3.03) and ABC transporter components *artI* (2.23 log_2_FC), *artM* (2.54 log_2_FC), and *artP* (4.55 log_2_FC) were also upregulated.

Genes involved in the glycine betaine biosynthesis pathway were highly expressed under salinity stress. The *betI* (transcriptional regulator; 3.51 log_2_FC), *betA* (3.97 log_2_FC) and *betB* (3.81 log_2_FC), showed notable upregulation. Additionally, glycine betaine transporters *opuAA* (5.10 log_2_FC) and *opuAB* (5.06 log_2_FC) were highly expressed, facilitating increased uptake of choline precursors.

### Regulation of oxidative phosphorylation pathway genes is altered under dual stress conditions

Several genes involved in the oxidative phosphorylation pathway were significantly enriched across three comparative groups: (i) 30 °C versus 35 °C at 60 ppt salinity, (ii) 30 ppt versus 60 ppt at 30 °C, and (iii) 30 ppt versus 60 ppt at 35 °C (Fig. 7 and Table S4). In particular, salinity stress at both temperatures induced the upregulation of genes related to ATP synthase. At 30 °C, *atpA* (2.03 log_2_FC), *atpC* (2.91 log_2_FC), *atpD* (2.02 log_2_FC), *atpF* (2.19 log_2_FC), and *atpG* (2.59 log_2_FC) were highly expressed. Similarly, at 35 °C, increased expression was observed for *atpC* (2.68 log_2_FC), *atpD* (2.14 log_2_FC), *atpF* (2.73 log_2_FC), *atpG* (2.54 log_2_FC), and *atpH* (2.46 log_2_FC). In contrast, *atpF* was notably downregulated (-3.87 log_2_FC) under higher temperatures with 30 ppt salinity.

In addition, genes encoding components of the electron transport chain were also upregulated under combined thermal and osmotic stress. For example, *sdhC*, part of Complex II, showed marked upregulation with 9.00 log_2_FC under 60 ppt salinity at 35 °C. Genes encoding cytochrome c oxidase Complex IV, including *ctaD*, *ctaG*, *ccoO*, and *ccoQ*, were also induced under high-salinity conditions at both 30 °C and 35 °C. These results align with the KEGG enrichment analysis, which identified oxidative phosphorylation (vha00190) as a significantly enriched pathway in the 30 ppt versus 60 ppt salinity comparison at 35 °C (Table 3). In contrast, temperature-induced activation of oxidative phosphorylation genes alone appeared relatively stable.

### Regulation of ribosomal protein genes under combined temperature and salinity stress

The expression of genes encoding 30S and 50S ribosomal protein subunits was markedly upregulated under high salinity at 35 °C, while remaining unchanged or downregulated under elevated temperature with normal salinity (Fig. 6 and Table S2). Specifically, under high salinity at 35 °C, 14 genes encoding 30S small ribosomal subunits and 19 genes encoding 50S large ribosomal subunits were significantly upregulated. Among these, *rpsJ*, which encodes the 30S ribosomal protein S10, displayed differential expression across all four comparative groups, with expression levels varying according to environmental conditions (Fig. 5). The highest *rpsJ* expression was observed under high salinity at 35 °C, with a log2 FC of 10.03, whereas the lowest expression (log2​FC of–3.72) occurred under 30 ppt salinity at elevated temperature (Table S3). These findings are consistent with KEGG pathway enrichment results for ribosomes (vha03010) under high salinity at 35 °C (Table 3).

### Temperature and salinity stresses influence the expression of genes associated with cell division and proliferation

The growth kinetics of *V. campbellii* were modulated by both temperature and salinity, as reflected in the expression patterns of genes associated with cell division and replication. RNA-seq analysis identified differential expression of several key genes aligned with the observed growth trends. Notably, *ftsA*, a gene essential for bacterial cell division, was significantly upregulated (3.29 log_2_FC) under elevated temperature combined with 60 ppt salinity. In contrast, both *ftsA* and *zapB* were markedly downregulated in the salinity comparison groups at both 30 °C and 35 °C, with log_2_FC values of − 6.53 and − 3.06, respectively.

### Regulation of *lux* operon genes under thermal and salinity stress conditions

Phenotypic analysis showed that elevated temperature led to a reduction in light emission, whereas increased salinity enhanced bioluminescence in *V. campbellii* (Fig. 2). Supporting this observation, RNA-seq data revealed the downregulation of multiple genes in the *lux* operon under thermal stress, particularly *luxC* (− 2.59 log_2_FC) and *luxD* (− 2.28 log_2_FC). In contrast, elevated salinity at 30 °C resulted in significant upregulation of *luxA*, *luxB*, *luxC*, *luxD*, and *luxE*, with log_2_FC values of 4.08, 4.18, 3.23, 4.69, and 3.72, respectively. At similar pattern was observed at 35 °C, where high salinity also induced the expression of *luxA* (2.85 log_2_FC), *luxB* (3.45 log_2_FC), *luxD* (3.25 log_2_FC), and *luxE* (2.12 log_2_FC).

### Temperature and salinity alter the expression of colonization and virulence-related genes in *Vibrio campbellii*

Host colonization by *V. campbellii* involves a complex interplay of physiological functions, including flagellar-driven motility, biofilm formation, and the activity of type secretion systems (TSSs). Our RNA-seq data corroborated phenotypic observations under combined temperature and salinity stress by revealing distinct gene expression profiles related to colonization traits. At elevated temperature in combination with 60 ppt salinity, multiple genes involved in flagellar assembly (*flgH, flgJ, filD, filM, filP, filS*) and flagellin synthesis (*flaA*, *flaB*, *flaD*, *flaE*, *flaF*, *flaG*) were downregulated (Fig. 6 and Table S2). Notably, *filP*, which encodes a component of the inner membrane flagella T3SS pore complex, was significantly repressed, with a log_2_FC of approximately − 7 under dual stress conditions. These findings are supported by KEGG pathway analysis, which identified significant enrichment of the flagellar assembly pathway (vha02040) under salinity stress (Table 3). In contrast, under increased salinity at 30 °C and 35 °C, these same flagellar genes were upregulated, aligning with enhanced motility observed phenotypically under these conditions.

Increased salinity also induced the expression of several genes implicated in biofilm formation. While genes directly responsible for exopolysaccharide production, such as transcriptional regulators *vpsT* and *vpsR*, did not show significant differential expression across the tested conditions, indirect biofilm-associated genes were notably upregulated under high salinity. In particular, the TRAP transporter genes (*dctPQM*), which are linked to TCA cycly intermediates and colonization capacity, were significantly expressed at both 30 °C and 35 °C. Additionally, *ttrS* gene, encoding a tetrathionate response regulator, showed substantial upregulation (up to 5.06 log_2_FC) under extreme stress (35 °C and 60 ppt salinity).

### Expression of virulence-associated genes in *Vibrio campbellii* is modulated by thermal and salinity stress

Our DEG analysis revealed significant downregulation of several virulence-related genes, particularly those linked to TSSs, under combined temperature and salinity stress (Fig. 6 and Table S2). Specifically, 28 genes encoding components of the T3SS were downregulated under high salinity at 35 °C. Among them, *exsE2*, encoding a T3SS translocation regulator, exhibited marked suppression, with log_2_FC values of − 5.65 and − 10.18 in the 30 °C versus 35 °C comparison at 60 ppt and in the 30 ppt versus 60 ppt comparison at 35 °C, respectively. Additionally, two T4SS-encoding genes (*traH* and *virB8*) and three T6SS genes (*impA*, *tagH*, and *tssJ*) were downregulated under increased salinity. Despite widespread suppression of TSS-related genes, some were upregulated. Notably, *epsL*, encoding a T2SS component, was strongly induced under various conditions, with log_2_FC values of 4.18, 7.38, and 3.18 in three comparisons: 30 °C versus 35 °C at 30 ppt, 30 ppt versus 60 ppt at 30 °C, and 30 ppt versus 60 ppt at 35 °C, respectively. Additional TTS-related genes, including *yscD*, *yscJ*, *cpaA*, *pilV*, and *hcp1*, also showed increased expression under high salinity.

Furthermore, many genes encoding extracellular virulence enzymes such as metallopeptidases and phospholipases were upregulated under high salinity at 30 °C and 35 °C. For instance, genes associated with peptidase activity showed stronger expression at 30 °C than 35 °C, with *cpg2*, *lasB*, and *pepT* upregulated at log_2_FC values of 4.05, 2.09, 2.51, respectively. Similarly, phospholipase-related genes, such as *volA*, showed increased expression (log_2_FC of 2.60 at 30 °C and 3.14 at 35 °C). In contrast, the *chiP*, encoding a chitoporin involved in chitinase transporter, was significantly downregulated under salinity stress, with log_2_FC values of − 3.07 and − 6.31 at 30 °C and 35 °C, respectively. Meanwhile, key virulence factors, such as *chiA* (chitinase A) and *hlyA* (hemolysin A) remained unchanged across all tested conditions.

### Correlation analysis between RNA-Seq and RT-qPCR data

To validate the DEGs results obtained from RNA-seq, 12 genes representing key functional categories, including ribosomal protein synthesis, colonization, transporter, motility, bioluminescence, and stress response, were selected for RT-qPCR analysis. Expression patterns were compared between the two datasets using Pearson’s correlation, with log_2_FC values calculated under the corresponding temperature and salinity conditions. The RT-qPCR results exhibited trends consistent with RNA-seq, reflecting similar directions of gene regulation, whether upregulation, downregulation, or no significant change (Fig. S3 A–D). Correlation coefficients (Pearson’s R) ranged from 0.69 to 0.97, indicating moderate to strong agreement between the two methods (Fig. S3E–F). These correlations were statistically significant (*p*-values ≤ 0.05), confirming the reliability of the RNA-seq datasets.

## Discussion

Our study demonstrates that temperature and salinity significantly influence both the genotypic and phenotypic traits of *V. campbellii* under controlled culture conditions. Notably, the pathogenic strain HY01 exhibited superior adaptability compared to the non-pathogenic strain, particularly at elevated temperatures (35 °C), even under suboptimal salinity conditions. While our study did not explore the adaptive mechanisms of *V. campbellii* under low salinity at high temperatures, this remains an intriguing avenue for future investigation. The findings provide valuable insights into the resilience of marine *V. campbellii* under combined temperature and salinity stress, which is relevant in the context of climate change and aquaculture settings.

At temperatures above 30 °C, a significant enhancement of virulence-related phenotypes was observed in both *V. campbellii* strains when salinity increased. The transcriptomic analysis corroborated these phenotypic observations, demonstrating that increased salinity enables *V. campbellii* to maintain several physiological functions, thereby enhancing its survival at elevated temperatures. Moreover, DEG profiles highlight stress response mechanisms involved in thermal and osmoadaptation, which facilitate bacterial survival and physiological resilience.

### Stress-responsive gene expression in *Vibrio campbellii*

Marine *Vibrio* species employ various defense strategies, particularly transcriptomic adaptation, to sustain essential cellular functions under environmental stress. USPs are key regulators involved in biofilm formation, adhesion, motility, virulence, and pathogenicity, enhancing bacterial survival under unfavorable conditions [23]. In this study, we observed upregulation of several USPs family genes (*uspABE*) in response to increasing salinity at 35 °C. These findings align with previous studies demonstrating high USP expression in *V. parahaemolyticus* exposed to crude bile [24], *Burkholderia cenocepacia* under low oxygen conditions [25], and *Listeria monocytogenes* during oxidative and acid stress [26]. Specific USPs are responsible for distinct phenotypic adaptations. For example, *upsA* is associated with bacterial growth and virulence during carbon starvation, elevated temperatures, oxidative stress, and host colonization [27, 28]. While *uspC* and *uspE* are involved in motility and adhesion, *uspD* regulates iron levels in *E. coli* during oxidative stress [29]. Our finding suggests that USPs serve as key protective elements in *V. campbellii*, supporting growth, motility, and biofilm formation under thermal and osmotic stress.

### Role of phage shock proteins and the CpxRA system in *Vibrio campbellii*

PSPs play a critical role in protecting bacterial cell membranes from environmental stress by maintaining cellular morphology and internal homeostasis [30]. Elevated temperature induced the expression of *pspACF* genes in *V. campbellii*, whereas salt stress suppressed their expression. PSPs help preserve membrane integrity and sustain proton motive force, essential for bacterial energy production [31]. This suggests that high temperatures act as a key trigger for PSP expression, ensuring cell envelope integrity and energy homeostasis in *V. campbellii*.

The two-component CpxRA system has been identified as an important regulatory mechanism activated in response to stress conditions, particularly those affecting the bacterial cell envelope. This system comprises two key protein components: the sensor kinase CpxA protein and the response regulator CpxR. The Cpx system is known to regulate multiple stress-responsive genes involved in cell envelope protection under various stressors [32]. Our DEGs analysis revealed overexpression of the *cpxA* gene under salinity stress but downregulation at higher temperatures with 30 ppt salinity. This finding suggest that high salinity acts as a stressor, inducing the expression of *cpxA*. Typically, upregulation of *cpxA* would be expected to influence *cpxR* expression, but in our study, *cpxR* expression was not detected under stress conditions. A previous study by Acosta et al. [33] demonstrated that the CpxRA system regulates genes encoding membrane-bound proteins involved in transport, energy metabolism, and cell membrane integrity in *V. cholerae* under low iron conditions. Consistently, this study observed upregulation of genes encoding membrane-localized proteins, particularly those involved in lipid transport and metabolism (e.g., *fadI*, *acs*). Conversely, some genes (e.g., *ppiA*, *cyoA*, *dgkA*) were downregulated, while others (e.g., *glsA*, *atpB*, *degP*), showed no expression, which corresponds with the absence of *cpxR* activation. Several of these membrane-responsive genes have been previously reported to be regulated by the CpxRA system in *E. coli* [34]. While the role of the CpxRA system has been well-documented in Gram-negative bacteria, its function in response to salt stress in *Vibrio* species, particularly in *V. campbellii*, remains unclear. Our study did not explicitly identify which genes are consequently regulated by the CpxRA system under salt stress. This presents an opportunity for further investigation into the role of the Cpx system in salt stress adaptation in *V. campbellii*. Our findings suggest that increased salinity induces osmotic pressure, leading to an imbalance of ion levels across the bacterial cell membrane, which can result in cell damage. Therefore, the upregulation of the *cpxA* gene, as a potential signal for *cpxR* induction, may consequently activate other genes involved in membrane protection, facilitating salt stress adaptation in *V. campbellii*.

### Thermal adaptation mechanisms in *Vibrio campbellii*

Bacterial stress-responsive programs typically activate the expression of specific genes to maintain cellular processes and molecular stability. The thermal-responsive gene *ibpA* was notably upregulated under combined thermal and salt stress. This finding aligns with a recent study indicating that IbpA functions as an initial preventive mechanism by restructuring protein complexes from large aggregates to smaller subunits, preventing protein misfolding and degradation under stress conditions [35]. Additionally, IbpA cooperates with other genes in temperature-responsive systems to maintain protein stability [36]. Our findings suggest that *ibpA* expression is induced not only by temperature shifts but also by salinity, contributing to cellular stability in *V. campbellii*.

Furthermore, our study identified the upregulation of two CSP-encoding genes, *cspV* and *cspD*, under extreme conditions. CSPs are typically recognized as cold-adaptive molecular components that help bacteria respond to sudden temperature downshifts. However, several studies have shown that CSP family proteins also response to various external stimuli, including oxidative stress, nutrient starvation, and antibiotic exposure in different bacterial species [37–39]. The primary function of CSPs involves nucleic acid stabilization, protein folding maintenance, metabolic regulation, and prevention of membrane fluid loss [40, 41]. A previous study reported that *cpsV* expression was induced when *V. cholerae* was exposed to bile salts [42]. Similarly, our findings align with a recent study demonstrating that *cspD* enhances bacterial growth at higher temperatures (28 °C) compared to extreme cold conditions (15 °C) in the fish pathogen *Aeromonas salmonicida* subsp. salmonicida [43]. Although the role of HSP and CSP family proteins in respond to various external inducers in *Vibrio* species remains incompletely understood, our results provide strong evidence that these genes contribute to protein homeostasis in *V. campbellii*. Our findings suggest that extreme environmental stress can trigger the upregulation of both HSP and CSP family proteins, thereby sustaining essential cellular processes and promoting bacterial survival. The expression patterns further suggest that salinity alone is insufficient to activate these stress responses. Instead, elevated temperature appears to function as the primary stressor, activating both heat and cold shock gene pathways to facilitate adaptation and survival under combined thermal and osmotic stress conditions.

### Osmoadaptation metabolic pathways in *Vibrio campbellii*

Salt-tolerant bacteria employ diverse strategies to maintain cellular homeostasis and energy production under salt stress. The “salt-out” strategy serves as a primary defense mechanism to regulate Na⁺ and K⁺ ion balance between intra- and extracellular levels [44]. In our study, genes encoding proton transporters were significantly upregulated, demonstrating that salinity stress triggers cation exchange through ionic transporters to prevent ionic toxicity and accumulation inside bacterial cells.

As a secondary defense mechanism, marine bacteria activate genes involved in key metabolic pathways associated with osmoprotectant production. Specifically, genes within the pyruvate pathway and tricarboxylic acid (TCA) cycle were significantly upregulated, supporting bacterial energy production under salt stress. The downregulation of the *pdhR* gene, a transcriptional repressor of pyruvate dehydrogenase complex (PDC), was observed in the pyruvate metabolic pathway. Suppression of *pdhR* may enhance the PDC activity by increasing the conversion of pyruvate to acetyl-CoA, a precursor for TCA cycle, thereby supporting energy generation in the respiratory metabolic pathway [45]. Within the TCA cycle, *sdhC* and *fumC* were significantly overexpressed, enhancing carbon utilization and energy production. Specifically, *sdhC* encodes succinate dehydrogenase, which generates energy in the form of FADH_2_ through the conversion of succinate to fumarate [46], while, the *fumC* gene encodes fumarase, which supports ATP synthesis by providing NADH during the conversion of fumarate to malate [47]. Interestingly, the glyoxylate cycle was activated at higher temperatures, serving as a metabolic shortcut for the direct conversion of acetyl-CoA to malate [48]. At 35 °C, the *aceB* gene, encoding malate synthase, was highly expressed, potentially enhancing malate production. Additionally, the upregulation of *dctPQM* genes, which encode a tripartite ATP-independent periplasmic (TRAP) transporter, suggests an increased import of C4-dicarboxylates, such as fumarate and malate, into the cell to support energy metabolism [49]. A previous study reported that salinity affects bacterial metabolic reprogramming, particularly influencing the TCA cycle, to ensure sufficient cellular energy for long-term survival in *E. coli* [50]. Additionally, environmental factors have been shown to impact TCA cycle activity, which can influence adhesion in *V. alginolyticus* [51]. The observed variations in genes related to the respiratory pathway and transport systems are significant findings of this study. These observations suggest two key roles of these gene expression changes: (i) ensuring an adequate supply of C4-dicarboxylates, which serve as crucial intermediates in carbohydrate metabolic pathways, and (ii) providing precursors (e.g., malate, glutamate) for the biosynthesis of compatible solutes that function as osmoprotectants under salt stress.

The biosynthesis and transport of compatible solutes (e.g., trehalose, glycerol, betaine, and proline) serve as a major osmoadaptive strategies in marine bacteria, including members of *Halomonadaceae*, *Natranaerobiaceae*, and *Vibrionaceae* families [44, 52]. This study observed the upregulation of multiple genes associated with the compatible solute system under high salinity conditions. Focusing on glutamate metabolism, we identified increased expression of *gltB*, which encodes glutamate synthase responsible for conversion of α-ketoglutarate and glutamine into glutamate. In contrast, *gltS*, a glutamate transporter gene, was downregulated, possibly indicating a regulatory mechanism to maintain intracellular glutamate balance. Upregulation of *proC* suggested enhanced conversion of glutamate to proline, a key osmoprotectant that helps prevent cellular damage. Concurrently, the *putA* gene was upregulated, potentially facilitating proline degradation back into the TCA cycle to support energy production [48]. The regulation of proline metabolism not only facilitates the production of compatible solutes for cellular protection but also contributes to maintaining adequate energy levels under salt stress. Additionally, the upregulation of genes involved in glutamate metabolism influenced the increased expression of *argH* and *artP*, which are part of the arginine metabolic pathway and utilize glutamate as a precursor for arginine biosynthesis. Moreover, temperature fluctuations appeared to modulate the expression of several metabolic pathways under salt stress. Notably, genes associated with cysteine and methionine metabolism were overexpressed at 35 °C, suggesting their potential role in osmoprotection. Conversely, biotin metabolism-related genes were predominantly upregulated at 30 °C. These findings suggest that *V. campbellii* employs alternative metabolic routes, such as those involving arginine, cysteine, and methionine, to enhance its resilience against osmotic and environmental stress [53]. The shift in metabolic strategy indicates that *V. campbellii* selectively activates pathways involved in compatible solute production while downregulating non-essential metabolic processes, thereby optimizing energy utilization and stress adaptation.

The regulation of *betAB* and *betI* genes, which are involved in the glycine betaine biosynthesis pathway, was highly induced under salt stress. The *betA* and *betB* genes facilitate the conversion of accumulated choline to glycine betaine, a key osmoprotectant that helps maintain osmotic balance within bacterial cells. Simultaneously, the upregulation of *betI*, which encodes a repressor regulatory protein for *bet*AB genes, suggests a potential mechanism to prevent excessive glycine betaine production, thereby maintaining intracellular osmolarity homeostasis. Additionally, we observed the upregulation of several transporter genes, particularly *opuAA* and *opuAB*, which encode the betaine-choline-carnitine-transporters (BCCT). These transporters likely enhance the uptake of choline, a precursor for glycine betaine biosynthesis pathway, further supporting osmotic adaptation [54]. Previous studies have also reported that glycine betaine biosynthesis is a major osmoadaptive strategy in various marine bacteria [55–57]. Our findings reinforce the critical role of glycine betaine biosynthesis in osmoadaptation, enabling *V. campbellii* to survive and thrive under high salinity conditions.

### Effects of thermal and osmotic stress on the expression of oxidative phosphorylation pathway genes in *Vibrio campbellii*

The oxidative phosphorylation pathway serves as a major energy-generating system in bacteria, linking carbohydrate and fatty acid metabolism to ATP production [58]. In this study, the significant enrichment of genes involved in oxidative phosphorylation underscores its essential role in the stress adaptation of *V. campbellii* under adverse environmental conditions. The ATP synthase complex, a key component of the electron transport chain, facilitates direct ATP production [59]. Our results showed that increased salinity led to the upregulation of several ATP synthase-related genes. These findings suggest that salinity serves as a primary stimulus for activating ATP synthase, ensuring rapid ATP synthesis and adequate energy supply for cellular metabolism.

Both temperature and salinity also influenced the expression of membrane-bound protein complexes involved in oxidative phosphorylation. Specifically, the *sdhC* gene, encoding the succinate dehydrogenase cytochrome b subunit, was significantly upregulated under elevated salinity at 35 °C. This gene is pivotal in the succinate pathway, linking the TCA cycle to oxidative phosphorylation by generating FADH_2_, to fuel the electron transport chain. In addition, several genes encoding components of the formate dehydrogenase complex and various cytochrome c oxidase subunits were also upregulated under high salinity conditions. These results suggest that salinity specifically induces an alternative energy production pathway, reinforcing a previous study demonstrating that temperature and salinity act as major regulators of oxidative phosphorylation genes in *V. alginolyticus* [60]. Elevated mRNA levels related to oxidative phosphorylation enhance carbon source utilization, facilitate compatible solute production, and contribute to the osmotic and thermal tolerance of *V. campbellii*.

### Enhanced expression of ribosomal protein-encoding genes in *Vibrio campbellii* under combined temperature and salinity stress

The ribosome pathway plays a crucial role in the translation of essential proteins, with bacterial ribosomes composed of 30S (small) and 50S (large) subunits [61]. In this study, DEGs related to ribosomal proteins were significantly enriched under higher salinity at 35 °C. Our findings are consistent with previous research indicating that environmental stressors influence ribosomal protein activity, implicating protein synthesis when bacteria experience osmotic or thermal stress [62]. In particular, salinity has been reported to induce ribosomal protein expression in marine bacteria such as *V. harveyi* [57]. Many ribosomal proteins exhibit functional interactions crucial for bacterial survival, enhancing stress adaptation and growth [63]. Among the DEGs related to ribosomal proteins, *rpsJ*, which encodes the 30S ribosomal subunit protein S10, showed the highest level of upregulation under high salinity conditions. This aligns with previous findings indicating that ribosomal protein S10 enhances growth and host colonization in *V. cholerae* [64]. Similarly, *rpsI* upregulation has been linked to bacterial fitness under salt stress [65]. Additionally, study in *E. coli* has demonstrated that ribosomal protein S10 functions as a feedback regulator, maintaining ribosomal assembly balance during non-limiting nutrient conditions [66]. However, the role of *rpsJ* in *V. campbellii* remains unknown. These results suggest that the enhanced transcription of ribosomal genes support the efficient transcription of stress-related proteins, facilitating *V. campbellii* adaptation to osmotic and thermal challenges. Notably, the coordinated upregulation of ribosomal and metabolic genes further highlights an integrated stress response mechanism, where ribosomal activity may contribute to sustaining alternative metabolic pathways under extreme environmental conditions. However, the differential expression of 30S and 50S ribosomal subunit genes alone does not fully elucidate the complex molecular processes underlying adaptation to combined temperature and osmotic stress. To validate these transcriptomic findings and gain deeper mechanistic insights, future studies should incorporate proteomic and post-translational analyses.

### Growth response of *Vibrio campbellii* to combined temperature and salinity conditions

Both temperature and salinity significantly impacted the growth of pathogenic and non-pathogenic *V. campbellii* strains. At 35 °C, the increase in salinity to 60 ppt promoted the growth of non-pathogenic ATCC BAA-1116 strain. However, this same condition adversely affected the growth of the pathogenic HY01 strain, suggesting a strain-specific adaptation strategy. Although extreme salinity levels modulated *V. campbellii* growth, they simultaneously enhanced key virulence-associated phenotypes such as bioluminescence, motility, and biofilm formation under heat stress. Supporting this observation, genes related to cell division (*ftsA*) were upregulated under high temperature and salinity. However, at extreme salinity levels, the expressions of *ftsA* and *zapB* (which regulate precise cell division and genetic material replication) were suppressed. These findings suggest that *V. campbellii* may downregulate cellular replication mechanisms as an energy-conservation strategy under extreme osmotic stress [67]. Despite this, our analysis revealed that increased salinity upregulated numerous genes involved in carbohydrate metabolism and oxidative phosphorylation, potentially compensating for growth limitations by boosting energy production. This suggests that salinity may act as a restorative growth factor, enhancing *V. campbellii* survival at high temperatures. However, at excessive concentrations, salinity could shift from being beneficial to detrimental, suppressing critical cellular processes, and affecting overall bacterial fitness.

### Temperature and salinity-mediated regulation of bioluminescence in *Vibrio campbellii*

Luminous *V. campbellii* typically harbors the *lux* operon, which governs its characteristic bluish luminescence. In this study, bioluminescence was significantly influenced by both temperature and salinity. Environmental factors such as temperature, pollutants, pH, and salinity can modulate the function of this operon [68]. Culture conditions not only affect luminescent intensity in *V. campbellii* but also in other luminous marine bacteria [69, 70]. Increasing the temperature to 35 °C resulted in delayed or inhibited light emission, correlating with the disrupted expression of *luxC* and *luxD*. Similar findings in *Photobacterium leiognathi* and *V. harveyi* suggest that elevated temperatures disrupt luciferase function and structure [71]. Consequently, heat stress hindered cellular energy production and bioluminescence, which has implications for shrimp aquaculture. At temperatures above 35 °C, detecting luminous *V. campbellii* in shrimp may become unreliable due to reduced bioluminescence. However, increased salinity restored luminescence. Several genes within the *lux* operon at 35 °C were upregulated, aligning with the increased luminescence observed in both HY01 and ATCC BAA-1116. This suggests salinity enhances bioluminescence at high temperatures. Similar observations in *V. harveyi* support this finding [57]. Our findings highlight temperature and salinity as critical factors shaping the physiological behavior of luminous marine bacteria.

### Effects of temperature and salinity on polar flagellum-driven motility in *Vibrio campbellii*

Swimming motility, driven by the polar flagellum, was disrupted under elevated temperature and salinity conditions. DEGs analysis revealed that several genes involved in flagellar assembly and flagellin synthesis were downregulated in response to elevated temperature. However, many of these genes were upregulated under increased salinity, indicating that salinity may partially compensate for thermal inhibition of motility. This finding is consistent with previous reports demonstrating that salt availability is essential for flagella-driven motility and energy production in marine bacteria [72], including *V. salmonicida*, where flagellin gene expression is also modulated by temperature and salinity [73].

Remarkably, expression of *fliP*, a key gene in the T3SS machinery essential for flagellar assembly [74], was markedly reduced under combined thermal and osmotic stress. This downregulation may impair the construction of the flagella T3SS channel, thereby affecting bacterial adhesion and colonization. Moreover, the broader DEGs profile revealed that temperature and salinity stress negatively impacted the expression of multiple genes encoding TSS proteins, further suggesting a compromised motility and virulence potential under dual-stress conditions.

### Combined effects of temperature and salinity on virulence gene regulation in *Vibrio campbellii*

The TSS system is a crucial virulence mechanism that enables bacteria to secrete degradative enzymes and toxins into the extracellular environment, facilitating host invasion [75]. In this study, many genes encoding TSS proteins, including T2SS, T3SS, and T6SS, were downregulated at higher temperatures and salinity, suggesting reduced virulence. Previous studies demonstrated that T6SS activity decreased with increasing temperature in *V. parahaemolyticus, V. fluvialis*, and *Pseudomonas plecoglossicida* [76–78]. Similarly, salt concentrations influence T3SS expression, with low salinity (0.5% NaCl) inducing its expression in *V. parahaemolyticus* [79]. However, different TSS proteins respond variably to environmental conditions. For example, in *V. parahaemolyticus*, T6SS1 (on chromosome I) is induced under marine salinity conditions below 37 °C, while T6SS2 (on chromosome II) is activated at temperatures below 30 °C with low salinity [80]. Despite the general suppression of TSS-related genes, our DEG analysis identified the upregulation of *epsL* under combined thermal and salinity stress. The *epsL*, which encodes a T2SS protein, is a key virulence factor responsible for secreting degradative enzymes such as chitinase and metallopeptidase, as well as biofilm matrix proteins in *V. cholerae* [81–83]. These results indicate that virulence-related gene expression in *V. campbellii* is modulated by environmental factors.

Extracellular enzyme production is another crucial virulence factor aiding pathogen invasion by degrading shrimp shell surfaces and host tissues. DEG analysis revealed upregulation of genes encoding chitinase, metallopeptidase, and phospholipase under higher salinity at 30 °C and 35 °C. These findings are consistent with previous studies showing that marine bacteria produce extracellular enzymes under a wide range of temperatures and salinity [84–86]. For example, *V. harveyi* produce chitinolytic enzymes at temperatures ranging from 16 to 37 °C [87]. Although chitinase production was not suppressed under stress conditions, the *chiP* gene, encoding an outer membrane chitoporin involved in chitinase uptake, was downregulated at higher salinity levels. This suggests that both temperature and salinity regulate the transcript-level expression of genes involved in enzymatic production.

### Salinity-induced enhancement of biofilm formation in *Vibrio campbellii* under thermal stress

Biofilms provide a protective environment for bacterial communities, aiding survival under adverse environmental conditions. Our findings indicate that elevated temperatures reduced biofilm formation in both pathogenic and non-pathogenic *V. campbellii* strains. However, increasing salinity, particularly at 60 ppt, enhanced biofilm formation and surface adhesion under thermal stress. This observation aligns with previous studies highlighting the role of biofilm formation in the environmental persistence of marine *Vibrio* species, such as *V. vulnificus*, *V. harveyi*, and *V. parahaemolyticus* [76, 88]. Transcriptomic analysis further corroborated the phenotypic findings, revealing temperature- and salinity-dependent regulation of genes associated with bacterial adhesion, colonization, and biofilm formation. Notably, genes associated with biofilm regulation were upregulated under combined thermal and osmotic stress conditions. In particular, the *dctPQM* gene cluster, encoding components of tripartite ATP-independent periplasmic (TRAP) transporters involved in the uptake of TCA cycle intermediates, was significantly upregulated. This enhanced expression may contribute to increased biofilm formation in *V. campbellii* under high salinity conditions. These findings are in line with previous findings in *V. cholerae* and *V. alginolyticus*, where TRAP transporters have been recognized as key virulence factors facilitating biofilm formation and host colonization [89–91]*.* Moreover, the regulatory gene *ttrS*, which encodes a tetrathionate response regulator, was also upregulated under dual-stress conditions. A previous study demonstrated that *ttrS* promotes *V. parahaemolyticus* colonization in murine models [92], suggesting its involvement in stress response and environmental persistence. Furthermore, several genes involved in flagellar function, TSS, and enzymatic production were also overexpressed, likely contributing to biofilm stability and structural integrity.

These findings suggest that increased salinity not only mitigates the negative effects of thermal stress on biofilm formation but also enhances bacterial adhesion and virulence potential. The co-regulation of genes involved in biofilm formation, motility, and secretion systems under these conditions highlights a coordinated stress response that facilitates the survival of *V. campbellii* in marine environments.

### Future research perspectives

This study elucidated the adaptive response mechanisms of *V. campbellii* under combined temperature and salinity stress using transcriptomic profiling. Phenotypic assays and transcriptomic findings demonstrate consistency between gene expression patterns and physiological responses. DEGs identified in this study provide a pool of candidate loci potentially involved in stress adaptation, particularly those related to metabolic processes, virulence regulation, and osmoregulation. However, functional validation of these genes is essential. Future studies incorporating targeted gene knockouts, CRISPR interference, and host infection models will be crucial to determine their specific roles in stress tolerance and pathogenicity. Importantly, this investigation focused solely on the pathogenic strain *V. campbellii* HY01, limiting the generalizability of findings due to potential genomic variability across strains. While the results offer valuable insights into strain HY01’s stress response, they also provide a foundational dataset for comparative transcriptomic studies among diverse *V. campbellii* isolates. Furthermore, the current transcriptomic analysis was conducted at a single time point under controlled laboratory conditions. Future work should incorporate time-course experiments and environmental simulations to explore dynamic gene expression changes under fluctuating marine conditions that better reflect natural ecosystems. These efforts will enhance our understanding of the ecological fitness and pathogenic potential of *V. campbellii* in response to ongoing ocean warming and salinity shifts.

## Conclusion

**Fig. 8:**
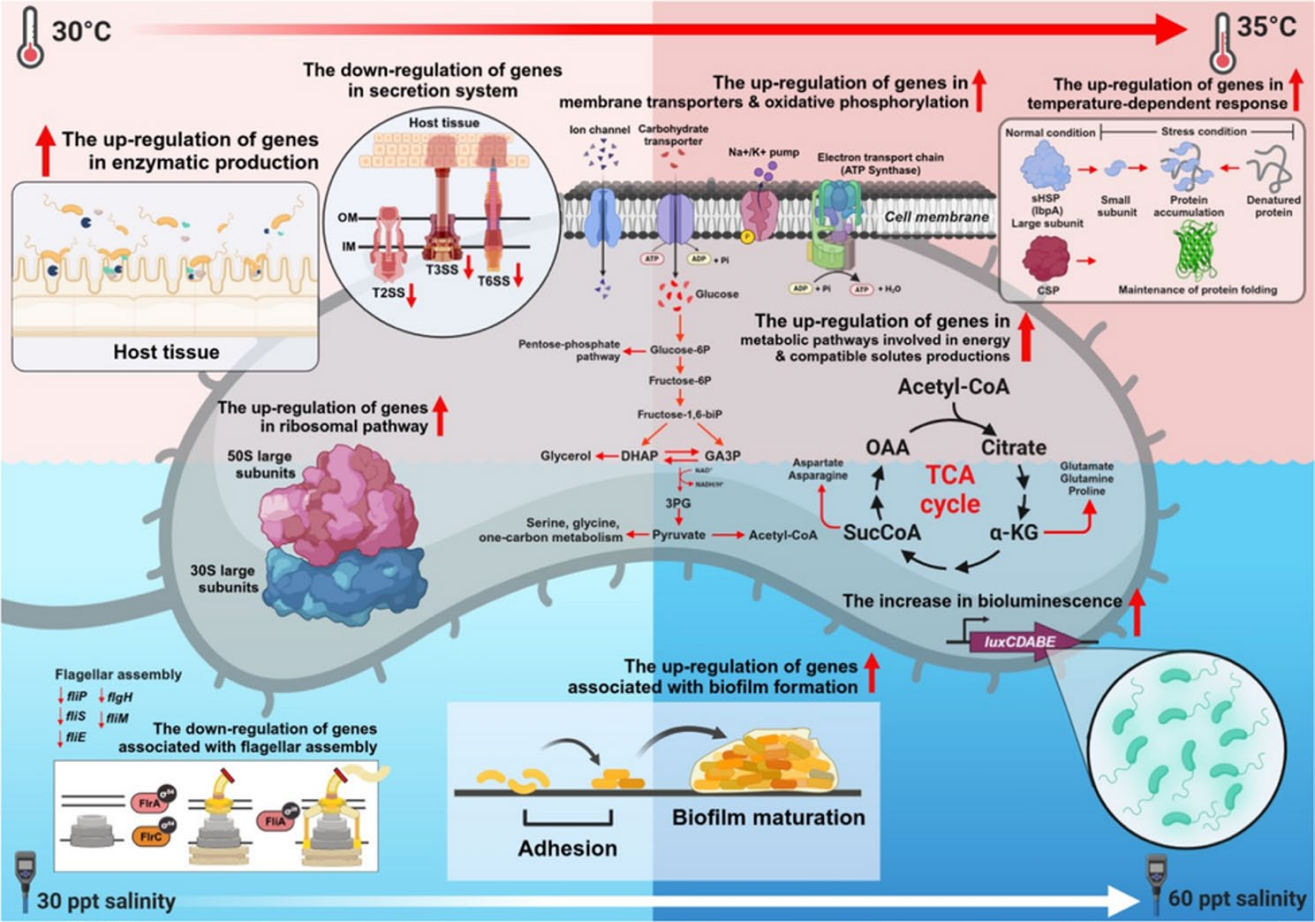
*Vibrio campbellii* adaptation to elevated temperature and salinity. The schematic illustrates key adaptive mechanisms used by *V. campbellii* under elevated temperatures and salinity conditions. *V. campbellii* utilizes oxidative phosphorylation for energy production, fueling carbohydrate metabolism and compatible solute pathways to enhance survival. Genes related to biofilm formation, adhesion, and virulence factors are upregulated, while secretion systems, bioluminescence, and motility genes are downregulated. Heat shock and cold shock proteins protect against protein misfolding and degradation, while ribosomal protein expression supports protein translation. Transporter systems and compatible solute pathways regulate ion homeostasis and prevent cell damage under osmotic stress. These adaptive responses enable *V. campbellii* to persist in high-temperature and high-salinity environments. This image was created using BioRender.com

Our RNA-seq analysis provides a comprehensive transcriptomic landscape of shrimp pathogenic *V. campbellii* exposed to dual temperature and salinity stress. Moving beyond primary phenotypic assessments, the DEGs uncovered in this study offer mechanistic insights into how this marine bacterium adapts at the molecular level. This work outlines an integrative model of stress adaptation (Fig. 8), revealing that salinity plays a crucial role in mitigating thermal stress through the induction of genes associated with metabolic pathways, oxidative phosphorylation, and ribosomal activity. Although combined stress conditions led to the downregulation of key virulence-related genes, such as those associated with T6SS and flagellar biosynthesis, *V. campbellii* compensates by upregulating genes linked to biofilm formation, energy production, and compatible solute biosynthesis. These findings suggest an adaptive transcriptional shift that supports survival and persistence in adverse marine environments. The study contributes to a broader understanding of how marine pathogens respond to anthropogenic environmental changes, such as rising sea temperatures and fluctuating salinity, factors increasingly relevant to microbial ecology and aquaculture disease management. Understanding the genomic basis of stress adaptations in *V. campbellii* is essential for predicting the impact of climate change on *Vibrio* outbreaks and guiding the development of mitigation strategies in shrimp aquaculture. Future research should explore the molecular interplay between environmental stressors and host–pathogen interactions to inform sustainable aquaculture practices in the context of global ocean change.

## Electronic supplementary material

Below is the link to the electronic supplementary material.

Supplementary Material 1

Supplementary Material 2

## Data Availability

The raw data presented in the part of transcriptomic analysis are available in the NCBI BioProject database under the accession number PRJNA1116105.

## Abbreviations

VBNC: Viable but non-culturable
LB: Luria–Bertani broth
LBS: Luria–Bertani broth supplemented with artificial sea salt
CV: Crystal violet
CLSM: Confocal laser scanning microscopy
SEM: Scanning electron microscopy
FITC-ConA: Fluorescein isothiocyanate-conjugated concanavalin A
PI: Propidium iodide
RNA-seq: RNA-sequencing
DEGs: Differentially expressed genes
FC: Fold change
FDR: False discovery rate
GO: Gene Ontology
COGs: Clusters of Orthologous Groups
RT-qPCR: Reverse transcription-quantitative PCR
TSS: Type secretion systems
BP: Biological process
CC: Cellular component
MF: Molecular function
USPs: Universal stress proteins
PSPs: Phage shock proteins
HSP: Heat shock protein
sHSP: Small heat shock protein
CSP: Cold-shock proteins
TCA: Tricarboxylic acid
PDC: Pyruvate dehydrogenase complex
ATP: Adenosine triphosphate
NADH: Nicotinamide adenine dinucleotide disodium salt
FADH_2_: Flavin adenine dinucleotide
TRAP: Tripartite ATP-independent periplasmic transporter
BCCT: Betaine-choline-carnitine-transporters
