# Supplementary material for: Genomic adaptations of Vibrio campbellii to thermal and salinity stress: insights into marine pathogen resilience in a changing ocean
Source: BMC Genomics. 2025 Aug 8;26:736. doi: 10.1186/s12864-025-11908-z (PMC12335131; doi:10.1186/s12864-025-11908-z)
Supplement: Supplementary file 1 — Supplementary Material 1 [file 12864_2025_11908_MOESM1_ESM.docx]

**

**

**Fig. S1** Functional enrichment analysis of differentially expressed genes (DEGs) assigned to Gene Ontology (GO) and Clusters of Orthologous Groups (COG) databases. The list of GO categories is divided into three domain containing biological process (BP), cellular component (CC), and molecular function (MF). The total GO categories associated with DEGs obtained from temperature comparative groups (30ºC vs 35ºC) under 30 ppt (A) and 60 ppt (B) salinity LBS. The top 10 GO categories associated with DEGs obtained from salinity comparative groups at 30ºC (E) and 35ºC (F). The bar plots depict the number of DEGs in GO sub-categories with a false discovery rate (FDR)-adjusted *P*-value ≤ 0.05. The number of DEGs mapped to COG categories is presented in the bar plots as follows: temperature comparative groups (30ºC vs 35ºC) under 30 ppt (C) and 60 ppt (D), and salinity comparative groups (30 ppt vs 60 ppt salinity LBS) at 30ºC (G) and 35ºC (H). The COG categories consist of chromatin structure and dynamics (B), energy production and conversion (C), cell cycle control, cell division, chromosome partitioning (D), amino acid transport and metabolism (E), nucleotide transport and metabolism (F), carbohydrate transport and metabolism (G), coenzyme transport and metabolism (H), lipid transport and metabolism (I), translation, ribosomal structure and biogenesis (J), transcription (K), replication recombination and repair (L), cell wall/membrane/envelope biogenesis (M), cell motility (N), post-translational modification, protein turnover, chaperones (O), inorganic ion transport and metabolism (P), secondary metabolites biosynthesis, transport and catabolism (Q), general function prediction only (R), signal transduction mechanisms (T), intracellular trafficking, secretion and vesicular transport (U), defense mechanism (V), extracellular structures (W), and function unknown (S). DEGs without assignment to the COG database are not included.

**
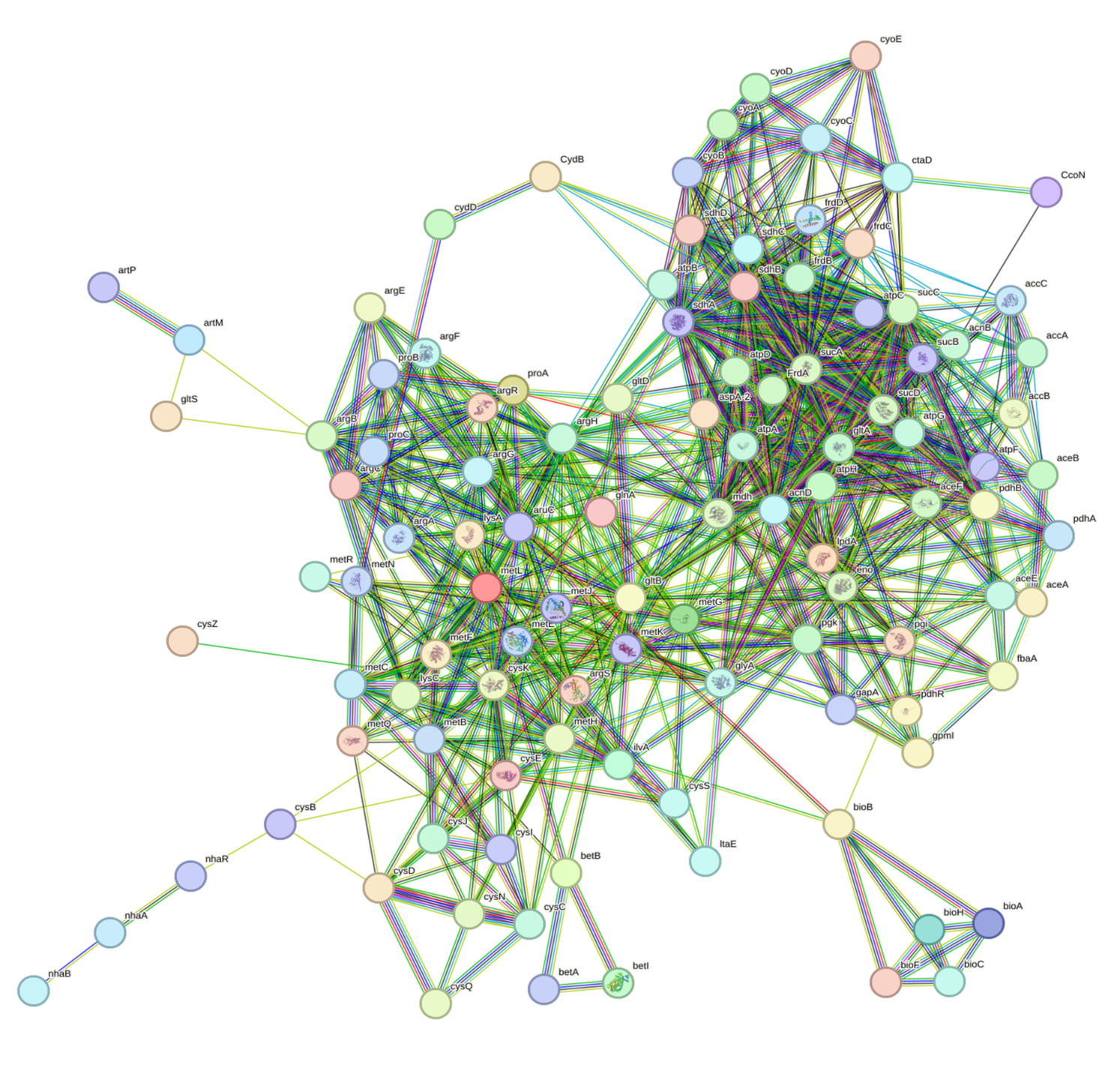
**

**Fig. S2** Interaction network of differentially expressed genes (DEGs) related to metabolic pathways, transporters, and oxidative phosphorylation. The different line colors of gene-to-gene connection represent predicted interactions, interaction databases, and additional information. Predicted gene interactions include gene neighborhood (
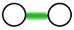
), gene fusions (
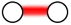
), and gene co-occurrence (
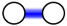
). The interaction databases comprise known interactions sourced from curated databases (
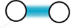
) and experimentally determined interactions (
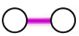
). Additional information includes associations identified through text mining (
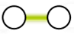
), co-expression (
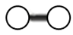
), and protein homology (
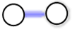
). Specific genes and their corresponding functions are detailed in Table S4 and mentioned in results and discussion section.

**
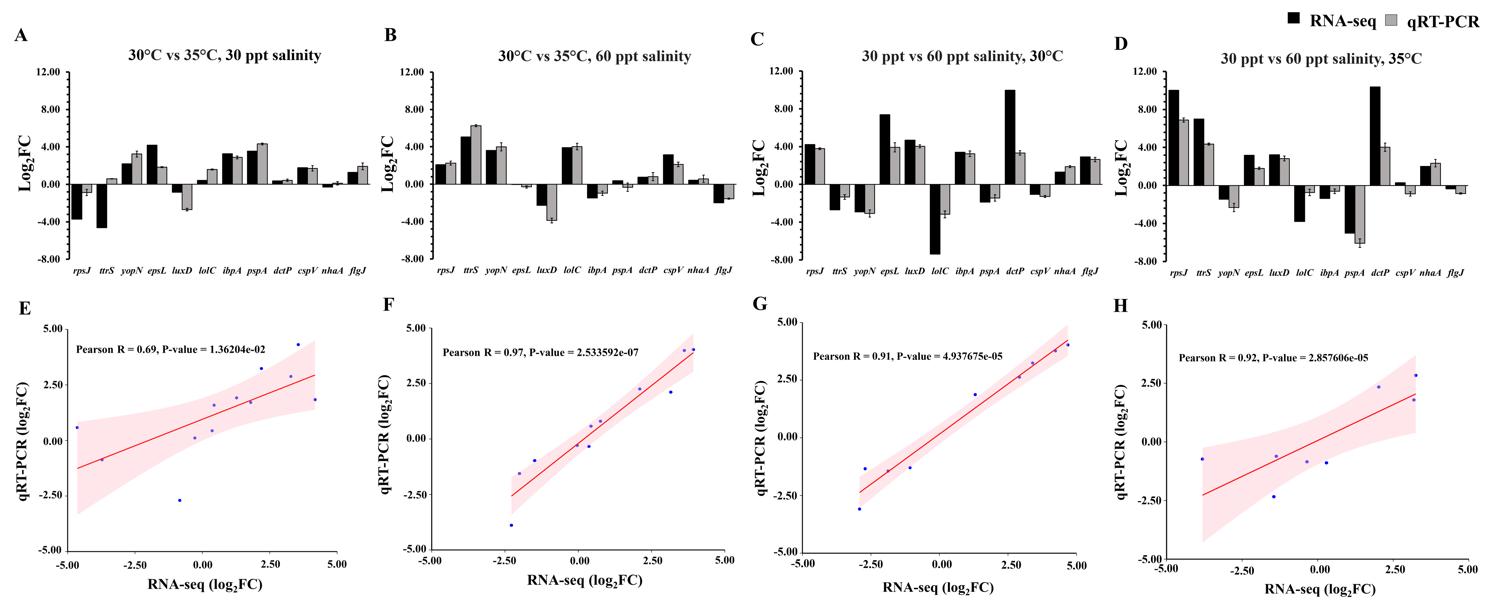
**

**Fig. S3** Comparison of gene expression profiles between RNA sequencing (RNA-seq) and quantitative real-time PCR (qRT-PCR). The scatter plot compares log_2_ fold change (FC) of selected adaptive genes in *Vibrio campbellii* HY01 under combined temperature and salinity conditions: **A** 30ºC vs. 35ºC under 30 ppt salinity, **B** 30ºC vs. 35ºC under 60 ppt salinity, **C** 60 ppt vs. 30 ppt salinity at 30ºC, **D** 60 ppt vs. 30 ppt salinity at 35ºC. Normalization of qRT-PCR was performed using the 16S rRNA gene. **E-H** Pearson’s correlation scatter plots comparing RNA-seq and qRT-PCR log_2_FC values across four conditions, showing a significant correlation between both datasets.
